# Selective expansion of cardiac macrophage subtypes distinguishes their functional roles in disease and homeostasis

**DOI:** 10.1172/JCI200194

**Published:** 2026-06-09

**Authors:** Rajesh K. Kasam, Ronald J. Vagnozzi, Yasuhide Kuwabara, Anne Katrine Z. Johansen, N. Scott Blair, Vikram Prasad, Suh-Chin J. Lin, Akanksha Rajput, Michelle Nieman, Jeffery D. Molkentin

**Affiliations:** 1 Department of Pediatrics, University of Cincinnati, Cincinnati Children’s Hospital Medical Center, Cincinnati, Ohio, USA.; 2 Division of Cardiology, Department of Medicine, University of Colorado Anschutz Medical Campus, Aurora, Colorado, USA.

**Keywords:** Cardiology, Immunology, Cardiovascular disease, Heart failure, Macrophages

## Abstract

Cardiac macrophages are broadly studied as 2 subtypes, tissue-resident CX3C chemokine motif receptor 1 positive (CX3CR1^+^) that are also CC motif chemokine receptor 2 negative (CCR2^–^) and monocyte-derived CCR2^+^. Previous systemic loss-of-function approaches suggested unique roles for each subtype in the heart, with CCR2^+^ being inflammatory and CX3CR1^+^ being prohealing. Here, we employed a cardiac-specific gain-of-function approach to selectively enhance either macrophage subtype. A robust increase in basal CCR2^+^ macrophages in the heart by targeted CC chemokine ligand 2 (*Ccl2*) expression did not induce inflammation, cause fibroblast activation, or impair cardiac function. However, increased CCR2^+^ macrophages reciprocally diminished self-renewing tissue-resident macrophages and worsened cardiac fibrosis due to pressure overload stimulation. Conversely, augmented expression of colony-stimulating factor-1 (*Csf1*) in the heart promoted selective expansion of resident CX3CR1^+^ macrophages, which exerted no pathophysiological consequences at steady state. However, pressure overload in these mice with expanded CX3CR1^+^ macrophages showed a CCR2^+^ macrophage–dependent inflammation leading to exacerbated cardiac dysfunction, simultaneously protecting from adverse remodeling and cardiac fibrosis. In conclusion, cardiac-specific selective enrichment of macrophage subtypes shows their intricate interplay and unique functional roles in regulating myocardial inflammation and fibrosis during hypertrophy and at homeostasis.

## Abstract 2

Cardiac macrophage subtypes were selectively expanded in the heart revealing their functional roles in disease and homeostasis

## Introduction

Macrophages are critical immune cells in the heart that are broadly classified into 2 subtypes based on their origin and function under homeostatic and pathological conditions (1–4). CX3C chemokine receptor 1–positive (CX3CR1^+^) tissue-resident macrophages are the predominant myeloid population in the healthy heart that originate during embryonic development that further consist of 2 distinct subpopulations defined by overlapping marker expression: T cell immunoglobulin and mucin domain-containing protein 4^+^ (TIMD4^+^) major histocompatibility complex class II^lo^ (MHC-II^lo^) and TIMD4^–^MHC-II^hi^ macrophages. TIMD4^+^MHC-II^lo^ macrophages are primarily maintained through self-renewal, while tissue-resident TIMD4^–^MHC-II^hi^ macrophages are partially replenished by blood-derived CC chemokine receptor 2–positive (CCR2^+^) monocytes that undergo transcriptional reprogramming (4–6). Recent studies have highlighted the role of these 2 subpopulations of tissue-resident macrophages in maintaining cardiac homeostasis, including supporting electrical conduction and cardiomyocyte health (7–9). In contrast, CCR2^+^ macrophages are scarce at steady state in the heart but robustly expand by external infiltration during cardiac stress such as ischemic injury or during more chronic injury states associated with hypertrophy and pressure overload–induced heart failure (10–12).

During cardiac stress leading to injury, macrophage populations undergo dynamic shifts that influence disease progression at both acute and chronic stages (13–17). In cardiac hypertrophy, CCR2^+^ macrophages are recruited, contributing to myocardial inflammation and fibroblast activation and fibrosis (18, 19). Studies using global cell depletion models or inhibition of CCR2^+^ macrophage recruitment have demonstrated functional benefits by reducing their activity, resulting in reduced fibrosis and improved cardiac outcomes with injury (3, 18, 20, 21). Conversely, systemic depletion or functional inhibition of CX3CR1^+^ resident macrophages have revealed their distinct contributions to augmenting healing through induction of angiogenesis and restraining tissue fibrosis and ventricular remodeling (4, 22–24). These tissue-resident macrophages are also important for long-term tissue homeostasis and the continual clearance and upkeep of the extracellular environment in the heart (7, 8). These past conclusions based on loss-of-function approaches and macrophage subtype depletion models also affected total systemic inflammation, potentially complicating the true heart-specific contributions of these subtypes.

Here, we demonstrate a system to preferentially increase either peripherally derived CCR2^+^ or tissue-resident CX3CR1^+^ macrophage content specifically in the heart by cardiomyocyte-specific ectopic expression of CC chemokine ligand 2 (*Ccl2*) or colony-stimulating factor-1 (*Csf1*), respectively. A robust increase in pro-inflammatory CCR2^+^ macrophages in an otherwise-healthy heart was not inherently pathological, but it did lead to augmented fibrosis after transverse aortic constriction–induced (TAC-induced) pressure overload. In contrast, substantial expansion of tissue-resident CX3CR1^+^ macrophages did not disrupt baseline cardiac structure, but with TAC stimulation it augmented total inflammation that worsened cardiac function. This effect was lost with depletion of CCR2^+^ macrophages. At the same time, increased CX3CR1^+^ macrophages restricted cardiac fibroblast activation and fibrosis after TAC stimulation, leading to protection from adverse remodeling.

## Results

### Macrophage subtypes in the mouse heart with aging.

**Figure 1:**
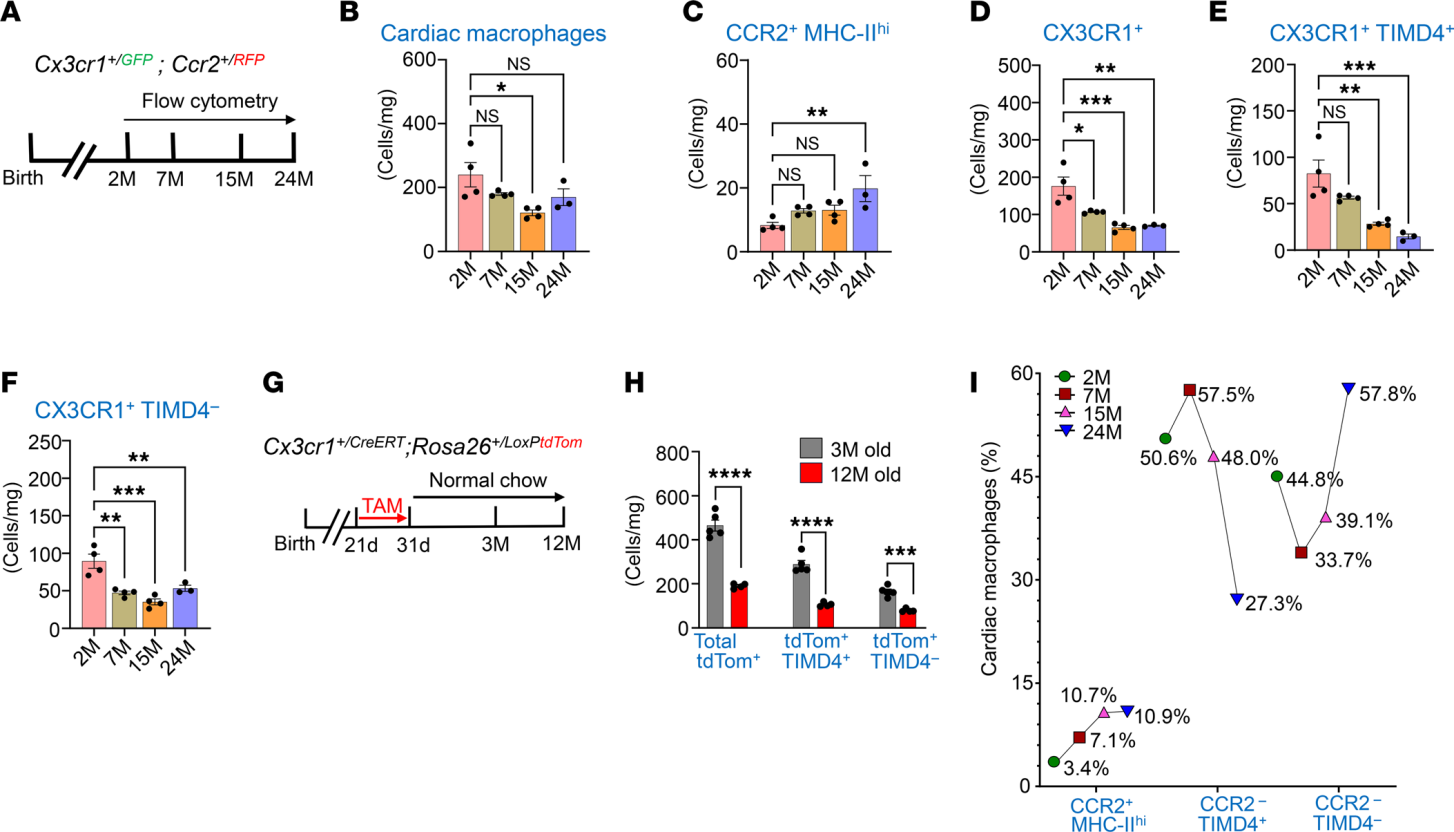
Dynamics of macrophage subtypes in the mouse heart with aging. (**A**) Experimental scheme showing different ages of *Cx3cr1^+/GFP^ Ccr2^+/RFP^* reporter mice from which cardiac immune cell populations were quantified from the heart using flow cytometry over the time shown in months (M). GFP, green fluorescent protein; RFP, red fluorescent protein. (**B**) Quantification of total cardiac macrophages flow-sorted as CD45^+^CD11b^+^CD64^+^ across the different ages and normalized to cardiac tissue mass. *n* = 3–4 mice per group, and error bars denote ± SEM. *P < 0.05 by 1-way ANOVA and Tukey’s multiple-comparison test. (**C**–**F**) Quantitation of total cardiac macrophages characterized as (**C**) CCR2^+^MHC-II^hi^, (**D**) total CX3CR1^+^ (CCR2^–^), and (**E** and **F**) CX3CR1^+^ and TIMD4^+^ or TIMD4^–^. *n* = 3–4 mice per group, and error bars denote ± SEM. *P < 0.05, **P < 0.01, ***P < 0.001 by 1-way ANOVA and Tukey’s multiple-comparison test. (**G**) Experimental scheme showing *Cx3cr1* lineage tracing mice (*Cx3cr1^+/CreERT^ Rosa26^+/tdTom^*) fed with tamoxifen (TAM) chow for 10 days starting at 3 weeks of age (21 days) followed by normal chow until 3 months or 12 months of age, and hearts were harvested for cardiac macrophage subtype quantification by flow cytometry. (**H**) Flow cytometry quantification of total *Cx3cr1* lineage-labeled cardiac macrophages (CD45^+^CD11b^+^CD64^+^CCR2^–^tdTom^+^) and subpopulations identified by TIMD4 marker expression in mice aged 3 months and 12 months. *n* = 4–5 mice per group, and error bars denote ± SEM. ***P < 0.001, ****P < 0.0001, by 2-tailed unpaired Student’s *t* test. (**I**) Graph showing percentage of total cardiac macrophages on the *y* axis quantified across different ages with flow cytometry parsed by cardiac macrophage subtypes based on CCR2 and TIMD4 expression on the *x* axis.

To investigate the dynamics of macrophage subtypes in the heart during aging, we used knock-in *Cx3cr1^+/GFP^ Ccr2^+/RFP^* macrophage reporter mice (hereafter “reporter mice”) and quantified these 2 populations via flow cytometry at 2 to 24 months of age (Figure 1A). The flow cytometry gating strategy (Supplemental Figure 1; supplemental material available online with this article; https://doi.org/10.1172/JCI200194DS1) excludes neutrophils (Ly6G^+^), monocytes (Ly6C^+^), and cluster of differentiation 11c–positive (CD11c^+^) dendritic cells. Total cardiac macrophages were identified based on CD45, CD11b, and CD64 expression (Figure 1B). Cell numbers were normalized to cardiac tissue mass, showing that total cardiac macrophages did not change from 2 to 24 months of age despite a slight reduction at 15 months (Figure 1B). The number of monocyte-derived macrophages (CCR2^+^MHC-II^hi^) in the heart progressively increased with aging, reaching significantly higher levels at 24 months compared with 2 months (Figure 1C). Conversely, the number of cardiac CX3CR1^+^ (which are CCR2^–^) macrophages significantly declined at 7 months, 15 months, and 24 months compared with 2 months of age (Figure 1D). Both CX3CR1^+^TIMD4^+^ and CX3CR1^+^TIMD4^–^ subpopulations showed reduced numbers across all ages when compared with 2 months (Figure 1, E and F). To more precisely assess the persistence of embryonic CX3CR1^+^ resident macrophages, we employed a *Cx3cr1* lineage tracing strategy using a pulse-chase design (25). Lineage tracing mice were administered tamoxifen containing chow for 10 days starting at 3 weeks of age to permanently label CX3CR1-expressing cells during this early window, followed by analysis at 3 and 12 months of age (Figure 1G). Flow cytometry quantification revealed a significant reduction in total tandem-dimer Tomato–positive (tdTom^+^) CCR2^–^ resident macrophages, both TIMD4^+^ and TIMD4^–^, at 12 months of age compared with 3 months (Figure 1H), showing a loss in embryo-derived cardiac-resident macrophage subtypes during aging.

To further evaluate the relative abundance of macrophage subtypes during aging, we adopted a simplified gating strategy based on CCR2 expression and divided total cardiac macrophages into CCR2^+^ and CCR2^–^ and then CCR2^–^ as TIMD4^+^ or TIMD4^–^ subpopulations as a percentage of the total cardiac macrophage pool (Figure 1I). At 2 months, the majority of cardiac macrophages were CCR2^–^ (~95%), while CCR2^+^ macrophages constituted only a small fraction (~3.4%). With aging, the proportion of CCR2^+^ macrophages progressively increased, reaching 10.9% by 24 months, consistent with prior studies showing the contribution of monocyte-derived macrophages to the total macrophage pool (1, 2). Notably, the proportion of CCR2^–^ macrophages that coexpress TIMD4 (25) was substantially reduced at 24 months of age compared with 2 months. In contrast, CCR2^–^ macrophages lacking TIMD4 expression declined in proportion at 7 and 15 months compared with 2 months, but increased thereafter, becoming the predominant CCR2^–^ population by 24 months (Figure 1I). These findings further suggest that embryonically derived resident macrophages are progressively lost and replaced by monocyte-derived cells that undergo phenotypic and transcriptional adaptation.

### Cardiac-specific CCR2^+^ macrophage enrichment in mice.

**Figure 2:**
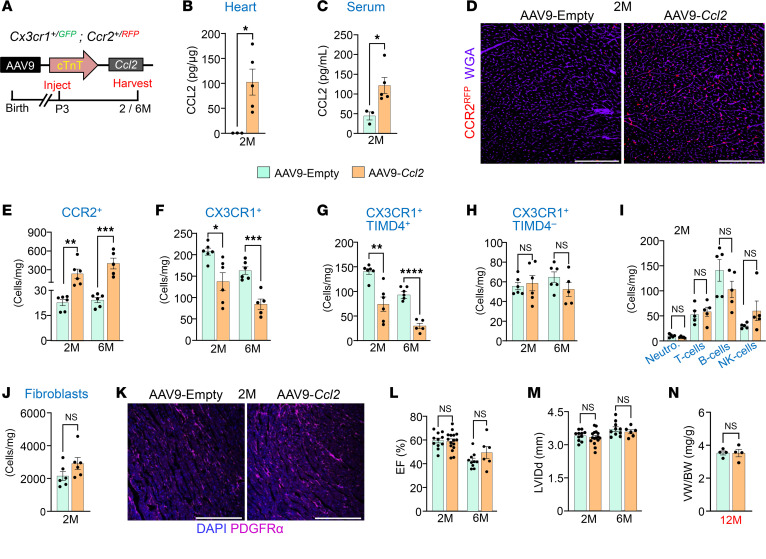
Cardiac-specific CCR2^+^ macrophage enrichment does not induce pathology in mice. (**A**) Experimental scheme showing AAV9-*Ccl2* vector injected into *Cx3cr1^+/GFP^ Ccr2^+/RFP^* (reporter) pups at postnatal day 3 (P3), with tissue harvested for analysis at 2 and 6 months of age. (**B**) Quantification of CCL2 cytokine levels by ELISA in cardiac tissue from AAV9-Empty– or AAV9-*Ccl2*–injected mice at 2 months of age. *n* = 3–5 mice per group, and error bars denote ± SEM. *P < 0.05, 2-tailed unpaired Student’s *t* test. (**C**) Quantification of serum CCL2 levels by ELISA in the indicated groups at 2 months of age. *n* = 3–5 mice per group, and error bars denote ± SEM. *P < 0.05, 2-tailed unpaired Student’s *t* test. (**D**) Representative immunofluorescence images of wheat germ agglutinin (WGA; membrane stain in purple) and RFP (red) to show CCR2^+^ cells from heart histological sections of the indicated groups of mice at 2 months of age. Scale bar = 200 μm. *n* = 4–5 mice per group. (**E**–**H**) Flow cytometry quantification of cardiac macrophage subtypes shown in each graph from the 2 groups of mice at 2 and 6 months of age by flow cytometry. *n* = 5–6 mice per group, and error bars denote ± SEM. *P < 0.05, **P < 0.01, ***P < 0.001, ****P < 0.0001, by 2-tailed unpaired Student’s *t* test. (**I**) Flow cytometry quantification of indicated immune cell populations in the heart from the 2 groups of mice at 2 months of age by flow cytometry (Neutro = neutrophils; NK = natural killer cells). *n* = 5 mice per group. Error bars denote ± SEM. Two-tailed unpaired Student’s *t* test. (**J**) Flow cytometry quantification of cardiac fibroblasts as CD45^–^CD31^–^MEFSK4^+^ from the hearts of the 2 groups of mice at 2 months of age. *n* = 6 mice per group, and error bars denote ± SEM. Two-tailed unpaired Student’s *t* test. (**K**) Representative immunofluorescence images of heart histological sections from the 2 groups of mice showing the fibroblast marker PDGFRα (purple) and nuclear stain DAPI (blue) at 2 months of age. Scale bar = 500 μm. *n* = 4 mice per group. (**L** and **M**) Echocardiography assessment of cardiac (**L**) ejection fraction percentage (EF%) and (**M**) left ventricular inner diameter at diastole (LVIDd) in the 2 groups of mice at 2 and 6 months of age. *n* = 6–16 mice per group. Error bars denote ± SEM. Two-tailed unpaired Student’s *t* test. (**N**) Ventricular weight to body weight (VW/BW) ratio from hearts of the 2 groups of mice at 12 months of age, injected at P3 with indicated viral vector. *n* = 4 mice per group, and error bars denote ± SEM. Two-tailed unpaired Student’s *t* test.

Here, we used a cardiac-specific model to ectopically express a cDNA for the *Ccl2* chemokine as a means of selectively increasing monocyte-derived CCR2^+^ macrophages chronically in the heart. We used recombinant adeno-associated virus 9 (AAV9) vector expressing *Ccl2* driven by the cardiac troponin T (cTnT) promoter to selectively recruit inflammatory macrophages to the heart (Figure 2A). We injected AAV9-Empty or AAV9-*Ccl2* in macrophage reporter mice (*Cx3cr1^+/GFP^ Ccr2^+/RFP^*) at postnatal day 3 (P3) (Figure 2A) and then quantified *Ccl2* mRNA and CCL2 protein levels in the heart (Supplemental Figure 2A and Figure 2B) and in the serum (Figure 2C) of these mice at 2 months of age. The increase in CCL2 at 2 months of age resulted in a ~20- to 30-fold increase in CCR2^+^ cells (RFP^+^) in the heart that was maintained through 6 months of age (Figure 2, D and E). The increase in serum CCL2 levels because of heart production of this cytokine did not increase monocyte-derived macrophages (RFP^+^CD68^+^) in quadriceps, liver, and lung of these mice as shown by immunofluorescence (Supplemental Figure 2B).

Interestingly, this 20- to 30-fold expansion of baseline CCR2^+^ macrophages in the heart with CCL2 overexpression led to a reciprocal reduction in cardiac tissue-resident macrophages at both 2 and 6 months of age as marked by CX3CR1^+^ and TIMD4^+^ (Figure 2, F and G) but not in CX3CR1^+^TIMD4^–^ macrophages that are typically derived from newly recruited CCR2^+^ cells (Figure 2H). This selective increase in CCR2^+^ monocyte-derived macrophages and reduction of tissue-resident macrophages was not accompanied by changes in neutrophils, T cells, B cells, and natural killer (NK) cells, suggesting a general lack of inflammation in the heart (Figure 2I). To determine if the reduction in TIMD4^+^ macrophages directly resulted from the increased presence of CCR2^+^ macrophages in the myocardium, we injected AAV9-Empty or AAV9-*Ccl2* into reporter (*Cx3cr1^+/GFP^ Ccr2^+/RFP^*) pups, as well as *Ccr2*-null pups (*Ccr2^RFP/RFP^*) that were characterized by monocytopenia and impaired CCR2^+^ monocyte recruitment into the heart (Supplemental Figure 2, C and D). Two months after AAV9-*Ccl2* injection, *Ccr2*-null mice showed significantly reduced CCR2^+^ macrophage presence in the myocardium compared with reporter mice (Supplemental Figure 2D), which then preserved TIMD4^+^ macrophage numbers (Supplemental Figure 2E). These results suggest that the recruitment of CCR2^+^ macrophages into the myocardium, but not *Ccl2* overexpression itself, impairs the TIMD4^+^ macrophage subpopulation in the heart, potentially due to niche competition within the myocardial microenvironment.

### Cardiac CCR2^+^ macrophages exacerbate TAC-induced fibrosis.

CCR2^+^ macrophages and tissue-resident CX3CR1^+^TIMD4^+^ macrophages can each directly influence the activity of fibroblasts and fibrosis in the heart but in opposite directions (4, 18). Interestingly, the ~20- to 30-fold expansion of CCR2^+^ macrophages within the heart due to CCL2 overexpression did not alter total cardiac fibroblast (CD45^–^CD31^–^MEFSK4^+^) content as observed by quantitative flow cytometry analysis and by immunohistochemistry for platelet-derived growth factor receptor-α–positive (PDGFRα^+^) cells assessed from cardiac histological sections (Figure 2, J and K). This expansion in inflammatory CCR2^+^ macrophages did not alter cardiac structure or function at 2 and 6 months of age, nor did it result in secondary hypertrophy by 12 months of age (Figure 2, L–N). Indeed, qRT-PCR analysis of extracellular matrix (ECM) gene expression in cardiac tissue (*Col1a1* and *Fn1*) or cardiac histological analysis of fibrosis by trichrome staining showed no differences between the 2 groups (Supplemental Figure 3, A and B). Moreover, histological analysis of fibroblasts (PDGFRα^+^) and CCR2^+^ macrophages (RFP^+^) in hearts of in AAV9-*Ccl2*–injected mice failed to show enriched direct interaction between these 2 cell types in vivo (Supplemental Figure 3C). This sustained increase in CCR2^+^ macrophages with CCL2 overexpression in the heart also failed to induce periostin expression in the heart over 12 months of age, again suggesting no effect on fibrosis or increased inflammation (Supplemental Figure 3D). Furthermore, single-nucleus RNA sequencing of hearts from 2-month-old control or AAV9-*Ccl2*–injected mice failed to show functionally distinct populations of inflammatory cell types in the heart, other than the known expansion of CCR2-expressing cells and reduction in TIMD4-expressing cells (Supplemental Figure 4, A–C). Moreover, qRT-PCR quantification of pro-inflammatory genes such as *Il6*, *Il1b*, and *Tnf* showed no differences in the hearts between the 2 groups of mice (Supplemental Figure 4D). These findings suggest that robust augmentation of CCR2^+^ macrophages in the heart, which drove a significant reduction in CX3CR1^+^TIMD4^+^ tissue-resident macrophages, was not pro-inflammatory or otherwise overtly detrimental to the heart.

**Figure 3:**
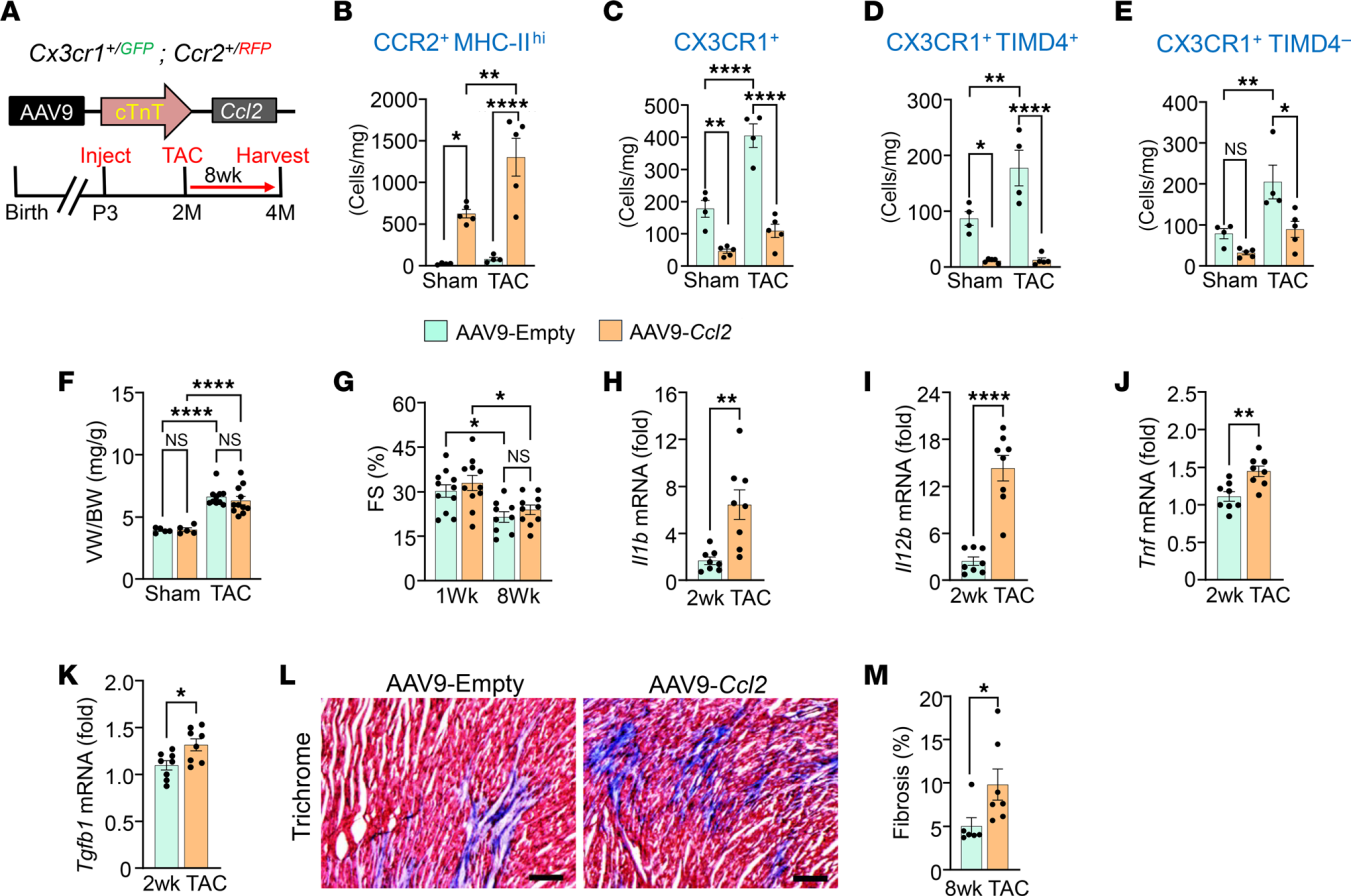
Increased cardiac CCR2^+^ macrophages exacerbate TAC-induced fibrosis. (**A**) Experimental scheme showing AAV9-*Ccl2* vector driven by cardiac troponin T promoter in AAV for injection in *Cx3cr1^+/GFP^ Ccr2^+/RFP^* reporter mice at P3 and then subjected to sham or TAC at 2 months of age and sacrificed for analysis at 4 months. (**B**–**E**) Quantification of macrophage subtypes from hearts of both groups of mice 8 weeks after sham or TAC as (**B**) CCR2^+^MHC-II^hi^, (**C**) total CX3CR1^+^, (**D**) CX3CR1^+^TIMD4^+^, and (**E**) CX3CR1^+^TIMD4^–^. *n* = 4–5 mice used in each group. Error bars denote ± SEM. *P < 0.05, **P < 0.01, ****P < 0.0001, by 1-way ANOVA with Tukey’s multiple-comparison test. (**F**) Ventricular weight to body weight ratio (VW/BW) 8 weeks after sham or TAC in both groups of mice. *n* = 5–11 mice per group. Error bars denote ± SEM. ****P < 0.0001, by 1-way ANOVA with Tukey’s multiple-comparison test. (**G**) Fractional shortening percentage (FS%) measured by echocardiography 1 and 8 weeks after TAC procedure. *n* = 9–11 mice per group. Error bars denote ± SEM. *P < 0.05, by 1-way ANOVA with Tukey’s multiple-comparison test. (**H**–**K**) qRT-PCR analysis of the indicated genes from the hearts of AAV9-Empty– and AAV9-*Ccl2*–injected mice and harvested 2 weeks after TAC. *n* = 8 mice per group, and error bars denote ± SEM. *P < 0.05, **P < 0.01, ****P < 0.0001, by 2-tailed unpaired Student’s *t* test. (**L**) Representative cardiac histology images with Masson’s trichrome staining for fibrosis (blue) in the 2 groups of mice 8 weeks post-TAC. Scale bar = 100 μm. (**M**) Fibrosis percentage as the blue-stained area quantified from these Masson’s trichrome histological sections. *n* = 6–7 mice per group and error bars denote ± SEM. *P < 0.05 by 2-tailed unpaired Student’s *t* test.

Recent studies observed that inhibiting the recruitment of CCR2^+^ monocyte-derived macrophages into the heart reduced left ventricular pathology and fibrosis after TAC in mice (18, 26). Here we hypothesized that the CCL2-based expansion of CCR2^+^ macrophages in the heart might hasten pathology in mice with TAC stimulation. We subjected AAV9-Empty– or AAV9-*Ccl2*–injected reporter mice to sham or TAC at 2 months of age, then analyzed the mice 8 weeks later (Figure 3A). AAV9-*Ccl2* robustly increased CCR2^+^ macrophages in the hearts of sham mice while reducing CX3CR1^+^TIMD4^+^ macrophages compared with AAV9-Empty controls (Figure 3, B–E). However, TAC stimulation for 8 weeks promoted an even greater CCR2^+^ macrophage expansion in the hearts of AAV9-*Ccl2* mice compared with controls (Figure 3B), but it did not further reduce CX3CR1^+^TIMD4^+^ macrophages (Figure 3, C–E). We also observed a significant increase in NK cells but not in T and B cells 8 weeks after TAC in the hearts of AAV9-*Ccl2*–injected mice compared with controls (Supplemental Figure 5, A–E). Unexpectedly, hypertrophy and cardiac function 8 weeks post-TAC were not exacerbated with greater CCR2^+^ macrophage content versus control (Figure 3, F and G). However, the presence of greater CCR2^+^ macrophages led to significant upregulation of myocardial pro-inflammatory and profibrotic genes such as *Il1b*, *Il12b*, *Tnf*, and *Tgfb1* after TAC (Figure 3, H–K), which were previously implicated in cardiac injury and fibrosis (18, 27, 28). There was also a significant increase in the cardiac fibrotic response with greater CCR2^+^ macrophage presence compared with control with 8 weeks of TAC as measured by Masson’s trichrome histological staining and quantitation (Figure 3, L and M). These results suggest that a selective increase in CCR2^+^ macrophages accompanied by reduced TIMD4^+^ content within the heart augments cytokine gene expression and the fibrotic response but without worsening cardiac systolic function or hypertrophy during chronic TAC-induced cardiac injury.

### Expansion of tissue-resident macrophages in the heart.

**Figure 4:**
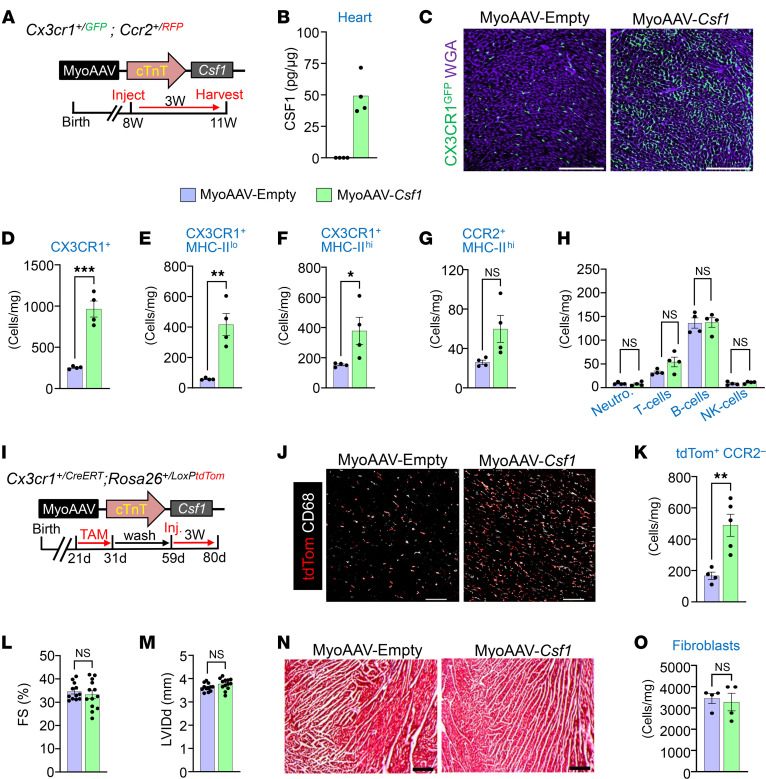
Controlled expansion of tissue-resident macrophages in the heart. (**A**) Scheme of the MyoAAV-*Csf1* recombinant MyoAAV expression system driven by the cardiac troponin T (cTnT) promoter injected retro-orbitally in adult reporter mice at 8 weeks of age and harvested 3 weeks later. (**B**) Cardiac tissue CSF1 levels measured by ELISA in the 2 groups of mice. *n* = 4 mice per group. CSF1 levels were not detectable in MyoAAV-Empty samples, but for visualization purposes it is shown as 0. (**C**) Representative immunofluorescence images from heart tissue sections of GFP (CX3CR1^+^) cells and WGA (membrane stain, purple) in the 2 groups of mice 3 weeks after control or MyoAAV-*Csf1* injection. *n* = 4 mice per group, scale bar = 200 μm. (**D**–**G**) Flow cytometry quantification from hearts of the indicated cardiac macrophage subtypes in both groups of mice shown as (**D**) CX3CR1^+^ macrophages, (**E**) CX3CR1^+^MHC-II^lo^ macrophages, (**F**) CX3CR1^+^MHC-II^hi^, and (**G**) CCR2^+^MHC-II^hi^. *n* = 4 mice per group, and error bars denote ± SEM. *P < 0.05, **P < 0.01, ***P < 0.001, by 2-tailed unpaired Student’s *t* test. (**H**) Flow cytometry quantification of the indicated immune cell populations from hearts of both groups of mice 3 weeks after MyoAAV injection (Neutro = neutrophils, NK = natural killer). *n* = 4 mice per group, and error bars denote ± SEM by 2-tailed unpaired Student’s *t* test. (**I**) Experimental scheme with tamoxifen (TAM) was given in *Cx3cr1* lineage tracing mice (*Cx3cr1^+/CreERT^ Rosa26^+/tdTom^*) for 10 days followed by 4 weeks of washout before injecting MyoAAV-Empty or MyoAAV-*Csf1*, followed by analysis 3 weeks later. (**J**) Representative immunofluorescence cardiac histological images of *Cx3cr1*-labeled (tdTom, red) macrophages also stained for CD68 (white) in mice 3 weeks after control or MyoAAV-*Csf1* injection. *n* = 4–5 mice per group. Scale bar = 100 μm. (**K**) Flow cytometry quantification of CX3CR1 labeled (tdTom^+^) CCR2-negative cardiac macrophages by flow cytometry, 3 weeks after MyoAAV injection. *n* = 4–5 mice per group. Error bars denote ± SEM. **P < 0.01 by 2-tailed unpaired Student’s *t* test. (**L** and **M**) Echocardiography assessment of cardiac fractional shortening percentage (FS%) and diastolic left ventricular dimension 3 weeks after control or *Csf1* vector delivery. *n* = 12–13 mice per group. Error bars denote ± SEM. Two-tailed unpaired Student’s *t* test. (**N**) Representative histological images of hearts for fibrosis (blue) with Masson’s trichrome staining 3 weeks after MyoAAV-Empty or MyoAAV-*Csf1* injection. Scale bar = 100 μm. (**O**) Cardiac fibroblast flow cytometry quantification as shown in Figure 2J from hearts of the 2 groups of mice. *n* = 4 mice per group and error bars denote ± SEM. Two-tailed unpaired Student’s *t* test.

Previous studies have utilized genetic or pharmacologic approaches to target *Csf1* or the Csf1 receptor (Csf1r) to deplete resident macrophages in tissues (11, 29, 30). To selectively increase cardiac-resident CX3CR1^+^ macrophage numbers in the adult mouse heart, we used the MyoAAV vector to express *Csf1* under the cTnT promoter to selectively drive expression in the heart (Figure 4A). MyoAAV-*Csf1* was delivered in 2-month-old reporter mice, and quantification of CSF1 levels 3 weeks later by ELISA showed an increase in cardiac tissue but not in serum (Figure 4B and Supplemental Figure 6A). The localized paracrine CSF1 signaling resulted in a substantial expansion in CX3CR1^+^ (GFP allele) resident macrophages in the myocardium, as assessed by immunostaining (Figure 4C). Flow cytometry quantification revealed 3- to 4-fold significantly greater numbers of total CX3CR1^+^ macrophages (Figure 4D), with both MHC-II^lo^ and MHC-II^hi^ (Figure 4, E and F) or TIMD4^+^ and TIMD4^–^ (Supplemental Figure 6, B and C) subpopulations showing significantly increased numbers. Notably, this resident macrophage expansion did not alter the number of CCR2^+^ monocyte-derived macrophages or other cardiac immune populations, including neutrophils, T cells, B cells, and NK cells, at baseline compared with controls (Figure 4, G and H). For all experiments, the MyoAAV-*Csf1* dosage was optimized to selectively increase CX3CR1^+^ resident macrophages by 3- to 4-fold without causing an increase in other immune cell types or CCR2^+^ macrophages in the heart.

To validate and extend these data, we performed tamoxifen-inducible *Cx3cr1* lineage tracing to track embryonically derived cardiac-resident macrophages (5, 25). *Cx3cr1^+/CreERT^ Rosa26^+/tdTom^* mice were given tamoxifen for 10 days to mark all *Cx3cr1* allele–expressing cells, including cardiac-resident macrophages and circulating monocytes, with tdTom^+^. A 4-week washout period was used so that only previously tdTom-labeled *Cx3cr1* lineage cells within the heart would be quantified (Figure 4I). The data showed that CSF1 directly expanded the tdTom-labeled cardiac tissue-resident macrophages, and the approach excluded recruitment of CCR2^+^ monocytes that could have converted to CX3CR1^+^ macrophages (Figure 4, J and K). Again, this basal increase in CX3CR1^+^ resident macrophages did not alter cardiac structure or function as measured by echocardiography (Figure 4, L and M), nor did it cause tissue fibrosis or cardiac fibroblast expansion, 3 weeks after MyoAAV-*Csf1* injection, compared with controls (Figure 4, N and O).

**Figure 5:**
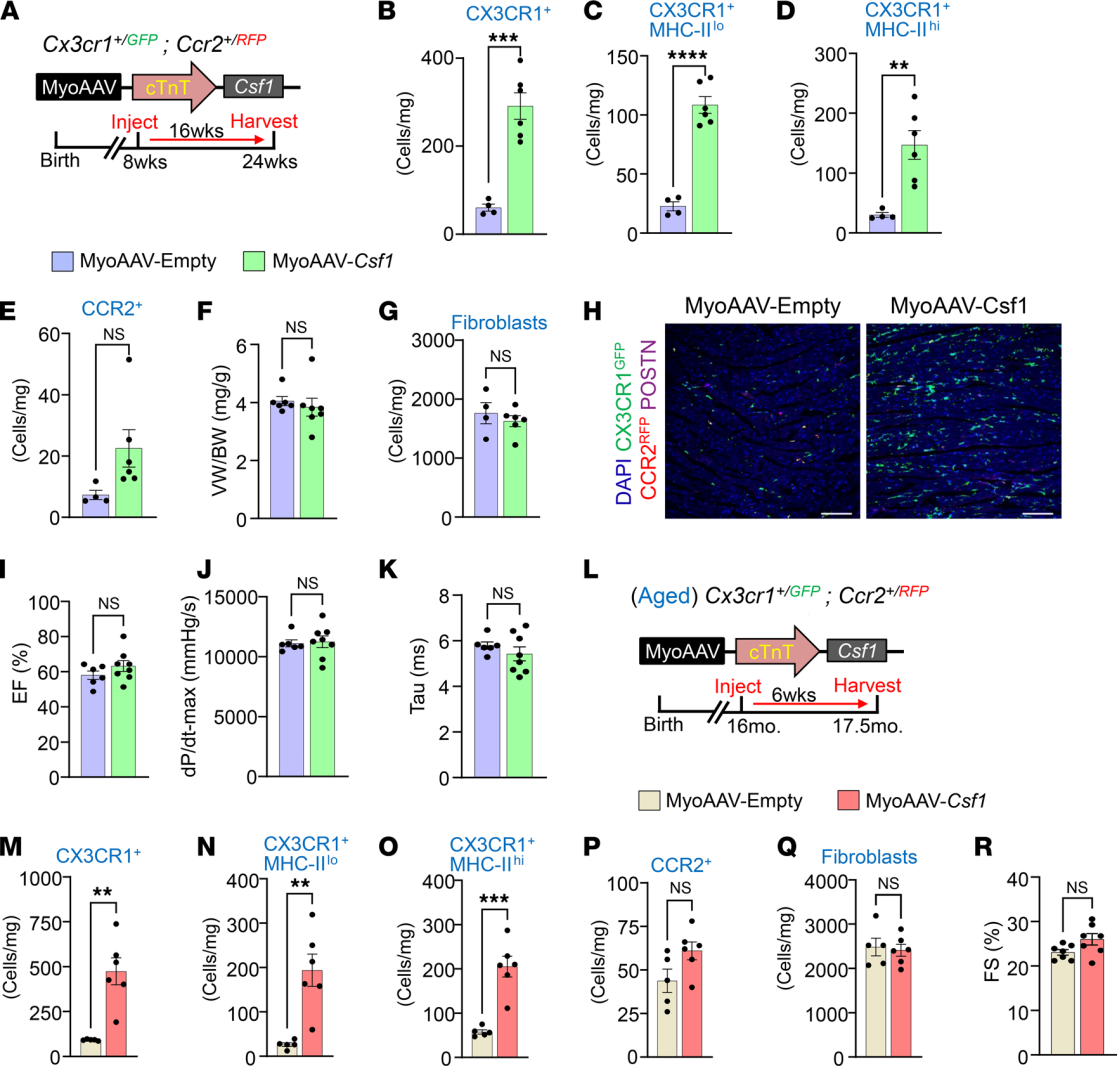
Long-term expansion of CX3CR1^+^ macrophages does not induce pathophysiological changes in the heart. (**A**) Experimental scheme showing MyoAAV-*Csf1* vector and experimental regimen with injection in adult reporter mice at 8 weeks of age and subsequent analysis 16 weeks later. (**B**–**E**) Flow cytometry quantification of cardiac macrophage subtypes in the indicated 2 groups of mice 16 weeks after MyoAAV injection: (**B**) total CX3CR1^+^, (**C**) CX3CR1^+^MHC-II^lo^, (**D**) CX3CR1^+^MHC-II^hi^, and (**E**) CCR2^+^ macrophages. *n* = 4–6 mice per group, and error bars denote ± SEM. **P < 0.01, ***P < 0.001, ****P < 0.0001, by 2-tailed unpaired Student’s *t* test. (**F**) Ventricular weight to body weight (VW/BW) ratio in the 2 groups of mice assessed at 16 weeks after MyoAAV injection. *n* = 6–7 mice per group, and error bars denote ± SEM. Two-tailed unpaired Student’s *t* test. (**G**) Cardiac fibroblast quantification by flow cytometry as shown in Figure 2J from hearts of the 2 groups of mice 16 weeks after MyoAAV delivery. *n* = 4–6 mice per group, and error bars denote ± SEM. Two-tailed unpaired Student’s *t* test. (**H**) Representative immunofluorescent heart histological images for DAPI (blue), CX3CR1 (green), CCR2 (red), and POSTN (purple) from the 2 groups 16 weeks after MyoAAV-Empty control or MyoAAV-*Csf1* injection. Scale bar = 100 μm. *n* = 4–6 mice per group. (**I**–**K**) Invasive hemodynamics measurement by cardiac catheterization was performed to measure (**I**) ejection fraction percentage (EF%), (**J**) *dP/dt* max, and (**K**) Tau at 20 weeks after MyoAAV-Empty or MyoAAV-*Csf1* injection. *n* = 6–8 mice per group, and error bars denote ± SEM. Two-tailed unpaired Student’s *t* test. (**L**) Experimental scheme showing MyoAAV-*Csf1* vector and injection in reporter mice at 16 months of age and analyzed 6 weeks later. (**M**–**Q**) Flow cytometry quantification of the indicated cardiac cells 6 weeks after control or MyoAAV-*Csf1* delivery as (**M**) total CX3CR1^+^, (**N**) CX3CR1^+^MHC-II^lo^, (**O**) CX3CR1^+^MHC-II^hi^, (**P**) CCR2^+^, or (**Q**) fibroblasts. *n* = 5–6 mice per group, and error bars denote ± SEM. **P < 0.01, ***P < 0.001, by 2-tailed unpaired Student’s *t* test. (**R**) Fractional shortening percentage (FS%) measured by echocardiography 6 weeks after MyoAAV-Empty control or MyoAAV-*Csf1* vector delivery in aged mice. *n* = 7 mice per group, and error bars denote ± SEM. Two-tailed unpaired Student’s *t* test.

We also examined reporter mice with 16 weeks of elevated CSF1 in the heart to examine longer-term effects (Figure 5A). MyoAAV-*Csf1* injection again significantly increased total CX3CR1^+^ macrophages by 3- to 4-fold (Figure 5B) and their subpopulations (Figure 5, C and D) but did not elevate monocyte-derived CCR2^+^ macrophages (Figure 5E), compared with controls. Transcriptomic data analysis of these mice revealed no change in the expression of cardiomyocyte-specific hypertrophic responsive genes, such as *Nppa*, *Nppb*, *Myh7*, *Myh6*, *Acta2*, *Acta1*, and *Tpm2*, or fibroblast-specific ECM genes, such as *Thbs4*, *Postn*, *Cthrc1*, *Col1a1*, *Ctgf*, *Fn1*, *Bgn*, *Col8a1*, and *Col14a1*, between control and MyoAAV-*Csf1*–injected mice (Supplemental Figure 6D). These results were further supported by an unchanged ventricle weight to body weight (VW/BW) ratio between the 2 groups (Figure 5F). Quantification of cardiac fibroblasts by flow cytometry showed no change (Figure 5G), nor did cardiac histological analysis of the fibrosis marker periostin (POSTN) show a change (Figure 5H), consistent with no increase in heart tissue fibrosis (Supplemental Figure 6E). Direct assessment of cardiac systolic and diastolic function by invasive hemodynamics with a pressure-transducing catheter also showed no pathological effects with 20 weeks of *Csf1* overexpression and a 3- to 4-fold expansion of tissue-resident macrophages in the heart (Figure 5, I–K). Finally, we also examined Evan’s blue dye uptake to assess cardiomyocyte necrosis and sarcolemma instability in these mice (Supplemental Figure 6F), which showed no difference between the 2 groups of mice.

Previous work identified a decrease in the proliferation rate of CX3CR1^+^ macrophages as a cause of age-related cardiac decline (2), and we observed reduced tissue-resident macrophage content in the aged mouse heart (Figure 1). Given these results we examined the effect of CSF1 on CX3CR1^+^ macrophage expansion in 16-month-old reporter mice (Figure 5L). Flow cytometry quantitation 6 weeks after MyoAAV-*Csf1* injection revealed significantly increased total CX3CR1^+^ cardiac macrophage number (Figure 5M) and their subpopulations (Figure 5, N and O) but not CCR2^+^ macrophages (Figure 5P). This increase in CX3CR1^+^ macrophages in the hearts of aged mice did not change cardiac fibroblast number versus control-injected mice (Figure 5Q), nor was there a deprecation in heart function measured by echocardiography (Figure 5R). Thus, 3- to 4-fold expansion of tissue-resident macrophages via CSF1 expression in the old or young mouse heart produced no pathological effect at baseline.

### CX3CR1^+^ macrophages worsen pathology without fibrosis after pressure overload.

**Figure 6:**
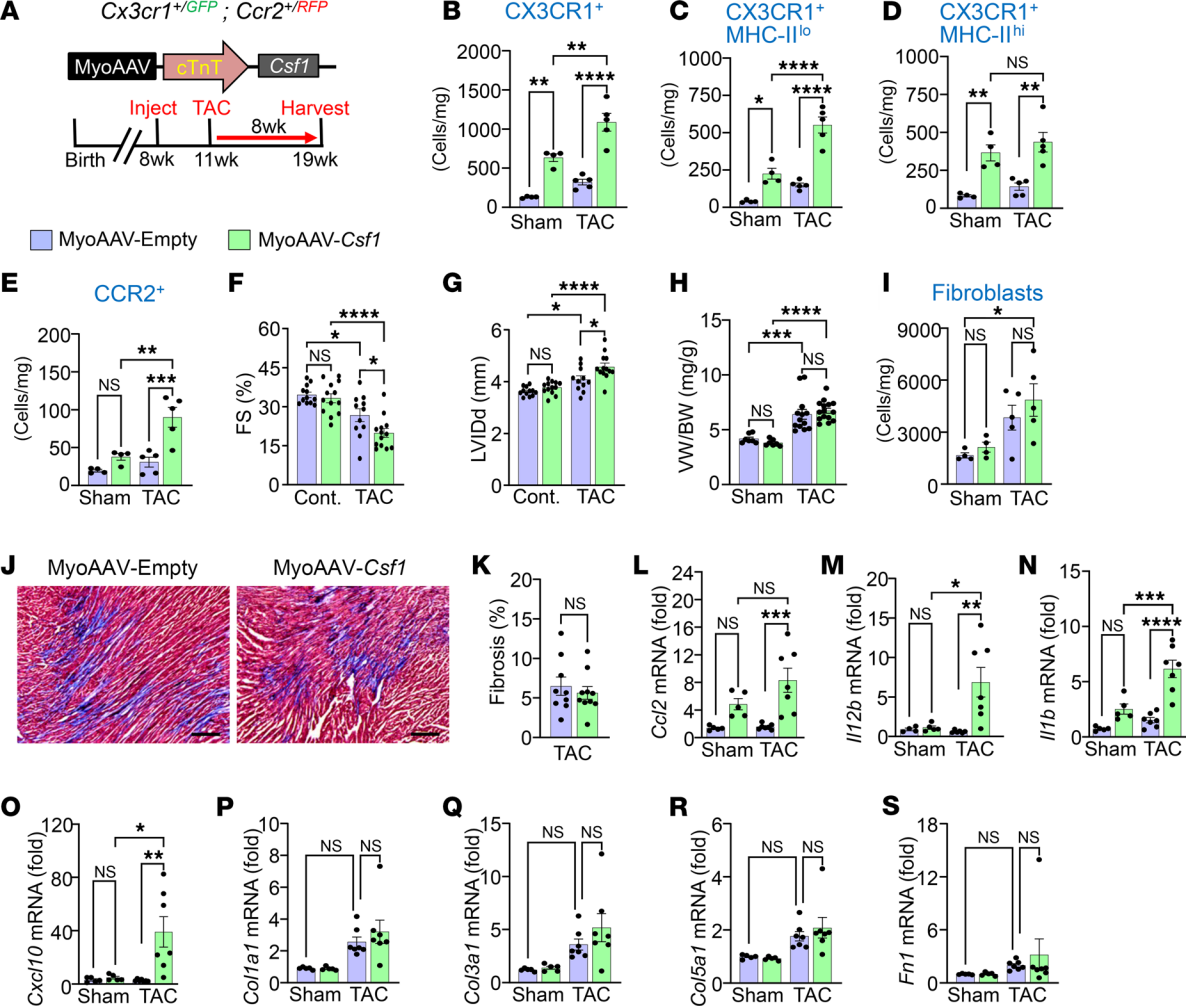
Augmented CX3CR1^+^ macrophages exacerbate cardiac dysfunction without fibrosis after pressure overload. (**A**) Experimental scheme showing MyoAAV-*Csf1* vector with injection in adult reporter mice at 8 weeks of age followed by TAC or sham surgery 3 weeks later and then harvesting 8 weeks later. (**B**–**E**) Flow cytometry quantification of macrophage subtypes in hearts of these 2 groups of mice as (**B**) total CX3CR1^+^, (**C**) CX3CR1^+^MHC-II^lo^, (**D**) CX3CR1^+^MHC-II^hi^, and (**E**) CCR2^+^ macrophages. *n* = 4–5 mice per group, and error bars denote ± SEM. *P < 0.05, **P < 0.01, ***P < 0.001, ****P < 0.0001, by 1-way ANOVA with Tukey’s multiple-comparison test. (**F** and **G**) Echocardiography in the indicated groups of mice 8 weeks after TAC to assess (**F**) fractional shortening percentage (FS%) and (**G**) left ventricular dimension in diastole. *n* = 11–13 mice per group, and error bars denote ± SEM. *P < 0.05, ****P < 0.0001, by 1-way ANOVA with Tukey’s multiple-comparison test. The control data without sham or TAC are from Figure 4, L and M. (**H**) Ventricular weight to body weight (VW/BW) ratio in the indicated 2 groups of mice 8 weeks after sham or TAC stimulation. *n* = 7–15 mice per group, and error bars denote ± SEM. ***P < 0.001, ****P < 0.0001, by 1-way ANOVA with Tukey’s multiple-comparison test. (**I**) Fibroblast quantification by flow cytometry from hearts of the 2 indicated groups of mice 8 weeks after sham and TAC. *n* = 4–5 mice per group. Error bars denote ± SEM. *P < 0.05, by 1-way ANOVA with Tukey’s multiple-comparison test. (**J**) Representative cardiac histology images with Masson’s trichrome staining for fibrosis (blue) in the 2 groups of mice 8 weeks post-TAC. Scale bar = 100 μm. (**K**) Fibrosis quantitation from Masson’s trichrome–stained cardiac histological sections in the 2 groups of mice 8 weeks after TAC stimulation. *n* = 9–10 mice per group, and error bars denote ± SEM. Two-tailed unpaired Student’s *t* test. (**L**–**S**) qRT-PCR analysis of the indicated genes from the hearts of MyoAAV-Empty– and MyoAAV-*Csf1*–injected mice 8 weeks after sham or TAC. *n* = 4–7 mice per group, and error bars denote ± SEM. *P < 0.05, **P < 0.01, ***P < 0.001, ****P < 0.0001, by 1-way ANOVA with Tukey’s multiple-comparison test.

To more thoroughly investigate the functional impact of increased cardiac tissue-resident macrophages on the heart, we again performed 8 weeks of TAC in adult mice with increased *Csf1* expression versus control mice (Figure 6A). Flow cytometry again revealed that CSF1 induced a significant expansion of total CX3CR1^+^ macrophages and their subpopulations compared with control-injected mice subjected to sham and TAC (Figure 6, B–D). TAC stimulation gave further expansion of CSF1-induced total CX3CR1^+^ and CX3CR1^+^MHC-II^lo^ macrophages as well as greater induction of CCR2^+^ macrophages (Figure 6, B–E). Additionally, total T cells, CD8^+^ T cells, and NK cells were increased in MyoAAV-*Csf1*–injected hearts compared with controls 8 weeks after TAC (Supplemental Figure 7, A–E). Interestingly, echocardiography assessment of cardiac structure and function showed significantly worse ventricular performance and greater ventricular dilation in mice with amplified CX3CR1^+^ tissue-resident macrophage content compared with controls (Figure 6, F and G), though both groups showed similar increases in cardiac hypertrophy (Figure 6H). Despite this greater pathological profile with TAC and *Csf1* overexpression, fibroblast numbers and fibrosis were not further affected (Figure 6, I–K), which is consistent with other studies showing that tissue-resident macrophages abate cardiac fibroblast activation with disease stimulation (4, 5, 11). However, qRT-PCR analysis revealed a significant increase in expression of pro-inflammatory genes including *Ccl2*, *Il12b*, *Il1b*, and *Cxcl10* in *Csf1*-overexpressing mouse hearts after TAC, compared with control (Figure 6, L–O), even though expression of fibrosis genes such as *Col1a1*, *Col3a1*, *Col5a1*, and *Fn1* was not induced to a greater extent versus controls (Figure 6, P–S). These findings indicate that despite elevated pro-inflammatory gene expression and worsened cardiac systolic function and remodeling with TAC stimulation, fibrosis and ECM gene expression remain unchanged in the presence of greater CX3CR1^+^ macrophage content, suggesting a negative regulation on fibroblast activity. By comparison, augmented CCR2^+^ heart content with *Ccl2* overexpression further augmented cardiac fibrosis with TAC stimulation even without reduced cardiac function (Figure 3, G, L, and M).

This potential negative regulation of fibroblast activity with TAC stimulation by CX3CR1^+^ macrophages is consistent with their spatial relationship in the heart assessed by immunohistochemistry. Here we observed that vimentin and PDGFRα-expressing (fibroblast markers) cells are enriched for direct contact with the CX3CR1^+^-expressing (GFP) cells in the heart (Supplemental Figure 8A). Reinforcing this concept, we observed a significant increase in fibroblast numbers in the hearts of 6-month-old *Cx3cr1*-null mice (*Cx3cr1*^GFP/GFP^) compared with age-matched controls (*Cx3cr1*^+/GFP^) at steady state, measured by flow cytometry (Supplemental Figure 8, B and C). Taken together, these data suggest that with TAC stimulation, greater CX3CR1^+^ macrophage content affects ventricular function in both positive and negative ways. Greater CX3CR1^+^ macrophage content augments a pro-inflammatory myocardial environment with TAC yet quells fibroblast activity and prevents excessive ECM accumulation.

To determine whether augmentation of CX3CR1^+^ macrophages confers benefit after the initial induction of hypertrophy, we delivered MyoAAV-Empty or MyoAAV-*Csf1* 1 week after TAC and analyzed 4 and 8 weeks after TAC (Supplemental Figure 9A and Supplemental Figure 10A). MyoAAV-*Csf1* delivery resulted in a significant expansion of total CX3CR1^+^ macrophages and subpopulations along with CCR2^+^ macrophages by 4 weeks after TAC (Supplemental Figure 9, B–F), which remained elevated at 8 weeks after TAC (Supplemental Figure 10, B–E). However, this increase in CX3CR1^+^ macrophages after TAC-mediated disease initiation did not further alter cardiac function, or hypertrophy, compared with control mice, despite greater myocardial inflammatory gene expression (Supplemental Figure 10, F–H and L–O), nor was there a difference in fibroblast number, cardiac fibrosis, and ECM gene expression between the 2 groups (Supplemental Figure 10, I–K and P–R). These results suggest that induction of CX3CR1^+^ macrophage expansion after TAC-mediated disease initiation is neither beneficial nor detrimental to disease outcome.

### Pathology by CX3CR1^+^ macrophages with pressure overload depends on CCR2^+^ cells.

**Figure 7:**
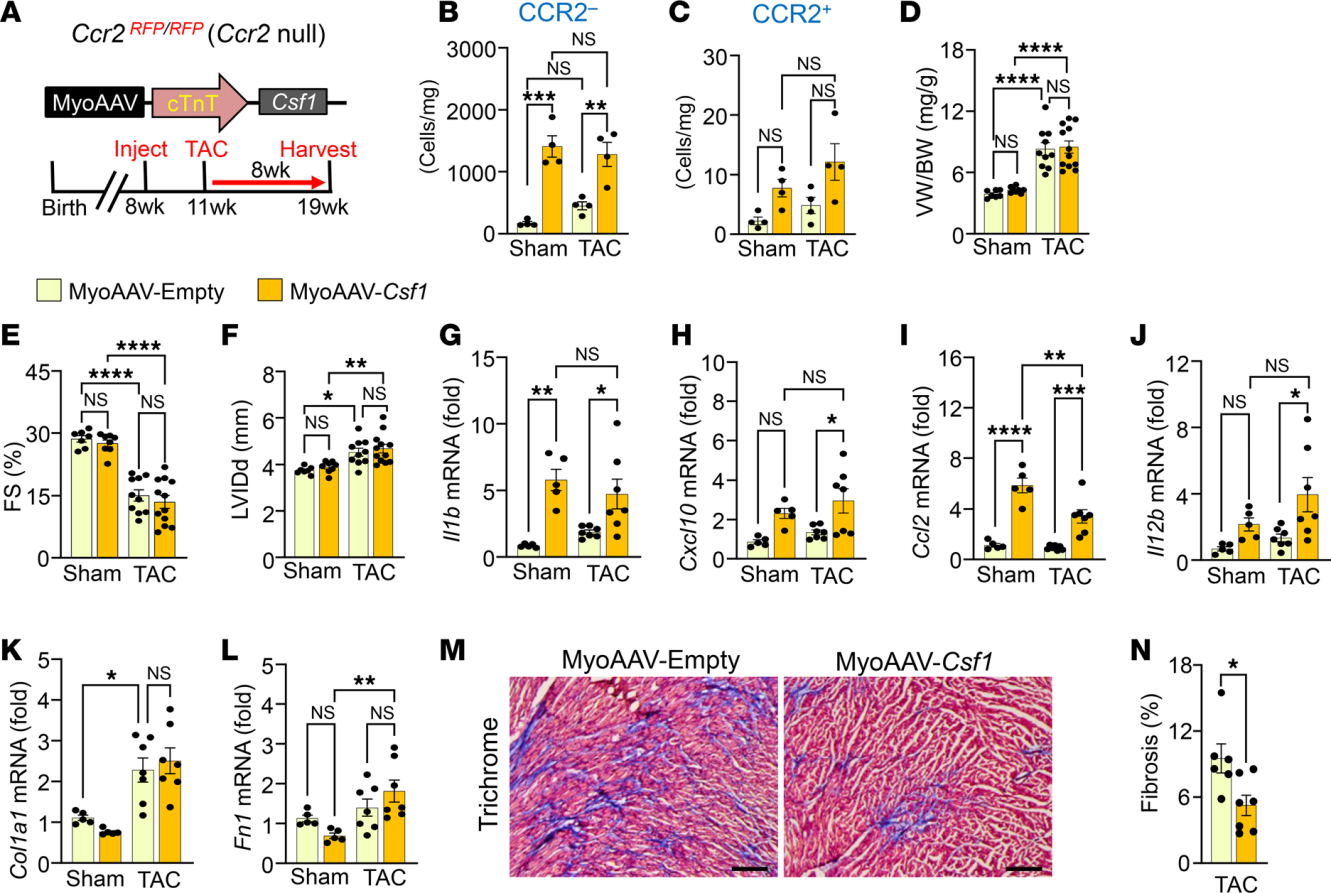
The negative impact of augmented CX3CR1^+^ resident macrophages with pressure overload depends on CCR2^+^ cells. (**A**) Experimental scheme showing MyoAAV-*Csf1* vector or empty vector control injection at 8 weeks of age in adult *Ccr2*-null mice (*Ccr2^RFP/RFP^*) and then subjected to sham or TAC 3 weeks later and harvested 8 weeks after that. (**B** and **C**) Flow cytometry quantification of (**B**) CCR2^–^ or (**C**) CCR2^+^ cardiac macrophages from hearts of the indicated 2 groups of *Ccr2*-null mice 8 weeks after sham or TAC. *n* = 4 mice per group, and error bars denote ± SEM. **P < 0.01, ***P < 0.001, by 1-way ANOVA with Tukey’s multiple-comparison test. (**D**) Ventricular weight to body weight (VW/BW) ratio in the indicated 2 groups of mice 8 weeks after sham or TAC. *n* = 7–12 mice per group. Error bars denote ± SEM. ****P < 0.0001, by 1-way ANOVA with Tukey’s multiple-comparison test. (**E** and **F**) Echocardiography in the indicated groups of mice subjected to sham or TAC to assess (**E**) fractional shortening percentage (FS%) and (**F**) left ventricular end diastolic dimension (LVIDd). *n* = 7–12 mice per group. Error bars denote ± SEM. *P < 0.05, **P < 0.01, ****P < 0.0001, by 1-way ANOVA with Tukey’s multiple-comparison test. (**G**–**L**) qRT-PCR analysis of the indicated genes from the hearts of MyoAAV-Empty– and MyoAAV-*Csf1*–injected mice after 8 weeks of sham or TAC. *n* = 5–7 mice per group and error bars denote ± SEM. *P < 0.05, **P < 0.01, ***P < 0.001, ****P < 0.0001, by 1-way ANOVA with Tukey’s multiple-comparison test. (**M**) Representative cardiac histology images with Masson’s trichrome staining for fibrosis (blue) in the 2 groups of mice 8 weeks after TAC. Scale bar = 100 μm. (**N**) Fibrosis percentage as the blue-stained area quantified from these Masson’s trichrome histological sections. *n* = 6–7 mice per group. Error bars denote ± SEM. *P < 0.05 by 2-tailed unpaired Student’s *t* test.

To investigate the role that CCR2^+^ cardiac macrophages might play in the pathological effects of CSF1-induced tissue-resident macrophage expansion, we utilized *Ccr2*-null mice (*Ccr2^RFP/RFP^*) injected with either MyoAAV-Empty or MyoAAV-*Csf1*, followed by sham or TAC procedures (Figure 7A). Compared with controls, hearts from MyoAAV-*Csf1*–injected mice again showed significantly increased CCR2^–^ macrophages in the hearts of both sham and TAC mice. However, TAC did not further augment CCR2^–^ macrophage number in *Ccr2*-null mice with MyoAAV-*Csf1* compared to sham (Figure 7B). Indeed, we observed low and unchanged CCR2^+^ macrophage levels in hearts of both control and MyoAAV-*Csf1*–delivered mice, regardless of sham or TAC procedure (Figure 7C). TAC again failed to differentially affect VW/BW ratio between control and MyoAAV-*Csf1* groups after TAC (Figure 7D). However, echocardiography assessment of cardiac structure and function now failed to show a greater detriment after TAC with CSF1-mediated resident macrophage expansion in *Ccr2*-null mice (Figure 7, E and F). Similarly, quantification of pro-inflammatory genes such as *Il1b*, *Cxcl10*, *Ccl2*, and *Il12b* (Figure 7, G–J) now showed no significant change between sham and TAC groups with MyoAAV-*Csf1* in *Ccr2*-null mice unlike previous data in reporter control mice that were not deleted for *Ccr2* (Figure 6, L–O). ECM gene expression (*Col1a1* and *Fn1*) continued to show no changes between control and MyoAAV-*Csf1* groups after TAC (Figure 7, K and L). To further investigate the pathological involvement of CCR2^+^ macrophages in CSF1-induced pathology, we performed transcriptomic analysis in 8-week post-TAC hearts from control reporter mice and *Ccr2*-null mice delivered with either MyoAAV-Empty or MyoAAV-*Csf1*. Expansion of CX3CR1^+^ resident macrophages that accompanied greater CCR2^+^ macrophages after TAC (as shown in Figure 6) resulted in a greater pro-inflammatory gene signature of innate immune cell–derived cytokines and Toll-like receptor signaling, a signature that was attenuated in *Ccr2*-deficient mice (Supplemental Figure 7F). Furthermore, augmented CCR2^–^ macrophage content in *Ccr2*-null mice prevented hearts from developing greater fibrosis 8 weeks after TAC compared with control MyoAAV–injected *Ccr2*-null mice (Figure 7, M and N), further emphasizing the antifibrotic function of cardiac-resident macrophages. Collectively, these results demonstrate that CCR2^+^ macrophages partly mediate the pathological effects of CSF1-induced CCR2^–^ macrophage expansion, ultimately exacerbating TAC-induced cardiac dysfunction through enhanced myeloid cell–driven inflammation.

## Discussion

Unexpectedly, our findings showed that selective increases in monocyte-derived CCR2^+^ macrophages in the heart did not negatively affect cardiac function or induce fibrosis under basal conditions. Even with chronic TAC stimulation the expansion of CCR2^+^ macrophages and the associated decline in tissue-resident macrophages did not impair cardiac function, although it did enhance a pro-inflammatory phenotype in the heart, culminating in increased fibrosis. This observation highlights the necessity for initiation of a broader pro-inflammatory signaling cascade to override the intrinsic homeostatic quiescent state of these CCR2^+^ recruited cells. Thus, the presence of substantially greater numbers of CCR2^+^ macrophages in the heart alone is not sufficient to initiate cardiac pathology without additional triggers (such as TAC stimulation). By comparison, expanding CX3CR1^+^ resident macrophages via CSF1 also did not negatively impact the heart at baseline in young and older mice, although with TAC stimulation there were augmented myocardial inflammation and worsened cardiac function that led to greater ventricular dilation but this time without exacerbating fibroblast activation and fibrosis. Moreover, chronic dysfunction of CX3CR1^+^ macrophages in *Cx3cr1*-null mice generated a progressive increase in fibroblast content in these hearts at 6 months of age, suggesting that this population of tissue-resident macrophages can directly regulate fibroblast activity in vivo. Additionally, CX3CR1^+^ macrophages likely exert antifibrotic functions partly by limiting CCR2^+^ macrophage–driven inflammation and pathology with TAC stimulation. This interaction is further underscored by the observation that, in the absence of CCR2^+^ macrophages (*Ccr2*-null mice), resident macrophage expansion now suppresses fibrosis and no longer leads to reduced cardiac function with TAC stimulation.

A previous study reported a significant loss of TIMD4^+^ resident macrophages following acute myocardial infarction was associated with a robust recruitment of CCR2^+^ monocytes in the adult heart (4, 24). We observed a corresponding decline in self-renewing TIMD4^+^ resident macrophages that was also associated with a sustained increase in CCR2^+^ macrophages in the myocardium even in the absence of injury. These collective observations suggest a potential direct competition between CCR2^+^ and TIMD4^+^ tissue-resident macrophages even in the absence of inflammation through a mechanism possibly involving myocardial niche competition.

Cardiac-specific enrichment of CCR2^+^ macrophages in the heart promoted greater fibrosis with TAC-stimulated hypertrophy. This effect was associated with increased expression of pro-fibrotic cytokines such as *Il1b*, *Tnf*, and *Tgfb1*, thereby potentiating ongoing inflammation and augmenting ECM deposition (27, 31). However, previous loss-of-function studies used a global monocyte depletion model that improved cardiac ventricular function along with reduced fibrosis in TAC-induced hypertrophy (18, 26), while our cardiac-specific gain-of-function approach that expanded CCR2^+^ macrophages showed no worsening of cardiac dysfunction with chronic TAC despite increased fibrosis. These differing results suggest that cardiac CCR2^+^ macrophages can drive fibrosis from within the heart in combination with reduced CX3CR1^+^ tissue-resident macrophage content but that effects of CCR2^+^ inflammatory macrophages from outside the heart are also likely to contribute to cardiac functional decline. These findings also underscore the importance of local shifts in cardiac macrophage subtype composition in dictating the response to disease stimulation from within the heart versus total multifactorial inflammatory disease states that can accompany select heart disease states.

We observed a decline in self-renewing, tissue-resident macrophages in aged mouse hearts, previously attributed to reduced proliferation efficiency (2). However, augmented CSF1 levels in the aged heart still led to a robust expansion of this tissue-resident macrophage population, suggesting that this subtype retains proliferative potential and that their decline with age may be due to a progressive loss of trophic signals such as CSF1. However, we speculate that long-term expansion and maintenance of greater tissue-resident macrophage content in the heart would likely protect from ongoing fibrosis but at the same time worsen other disease and inflammatory processes. Indeed, this predisposition to general inflammatory remodeling was inhibited in the CX3CR1^+^ expanded hearts by blocking the inflammatory effects of CCR2^+^ cells or by expanding them after disease initiation. Consistent with this interpretation, a recent study demonstrated that alternatively activated cardiac-resident macrophages, which were primarily CCR2^–^, contributed to pathology via enhanced IL-4Rα signaling during chronic ischemic cardiomyopathy (32). Thus, it remains unclear how best to alter cardiac macrophage subtypes long term in a way that would be exclusively protective, as it is also true that macrophage activity and the ability to selectively augment even CCR2^+^ cells can be adaptive and protective, such as for insulation of conducting areas of the heart (7) and continual removal of extracellular materials under baseline homeostasis conditions (8) or with acute injury (33). However, the unexpected results from our study are that expansion of either of these 2 primary macrophage subtypes in the heart can have both positive and negative ramifications, suggesting the need for greater temporal regulation toward achieving a therapeutic advantage that depends on the local and systemic disease processes in play.

## Methods

### Sex as a biological variable.

We included both male and female mice in our study in similar ratios. Our findings were similar for both sexes, indicating that the reported results were not sex specific. However, to exclude an influence of sex on the transcriptional profile with limited sample number, only males were selected for bulk RNA-sequencing (Supplemental Figure 6D). For qRT-PCR analysis, a combination of male and female mice was utilized.

### Animals.

All animal experiments were approved by the Institutional Animal Care and Use Committee of the Cincinnati Children’s Hospital Medical Center (Protocol IACUC 2025-0047). All the mouse lines were available commercially and were purchased from The Jackson Laboratory (Jax). To generate *Cx3cr1^+/GFP^ Ccr2^+/RFP^* mice, homozygous *Cx3cr1^GFP^* knock-in mice (*B6.129P-Cx3cr1tm1Litt/J*, Jax no. 005582) were mated with homozygous *Ccr2^RFP^* knock-in mice [*B6.129(Cg)-Ccr2tm2.1Ifc/J*, Jax no. 017586]. Homozygous *Cx3cr1^GFP^* knock-in mice or homozygous *Ccr2^RFP^* knock-in mice served as global nulls for their respective gene. For *Cx3cr1* conditional macrophage lineage tracing, homozygous *B6.129P2(Cg)-Cx3cr1^tm2.1(cre/ERT2)Litt^/WganJ* (Jax 021160) mice were mated with homozygous *B6.Cg-Gt(ROSA)26Sor^tm14(CAG-tdTomato)Hze^/J* (Jax 007914) mice to yield double heterozygous *Cx3cr1^+/CreERT^ Rosa26^+/tdTomato^* mice that were then fed with tamoxifen-containing chow (Envigo TD.130860) as we have published previously (34). The tamoxifen administration experiments with washout are described in the experimental schemes shown in Figure 1G and Figure 4I. For experiments in wild-type mice, *C57/Bl/6J* (Jax 000664) were used. Both male and female mice were used for experiments unless indicated otherwise, at the age indicated in the figure legend or text for each experiment. Mice were housed in a specific pathogen–free, temperature-controlled vivarium under a 12-hour light/dark cycle with ad libitum access to food and water. Mice were allocated numbers that allowed blinding during the surgical procedure, cardiac function analysis, single-nucleus sequencing, and bulk RNA transcriptomic analysis.

### pAV-cTnT-Ccl2 and pAV-cTnT-Csf1 generation.

Mouse cDNA for *Ccl2* was custom-generated and inserted into the pUC57 vector by BioBasic. The insert was PCR-amplified with primers containing an NheI restriction site (forward) and an Mlul restriction site (reverse). The destination, pAV-cTnT plasmid (PM10013, Vigene Biosciences), and the PCR fragment were digested with NheI and Mlul and ligated together with T4 DNA ligase (Thermo Fisher Scientific). The full-length open reading frame of mouse *Csf1* was PCR-amplified with primers incorporating an EcoRI site and Kozak sequence upstream (forward) and NheI/SalI sites downstream (reverse). The PCR product was digested with NheI and ligated (ligation kit, Takara) into the pAV-cTnT expression vector that contains the cTnT promoter, linearized with BglII (blunted) and NheI. All restriction enzymes were purchased from New England Biolabs. The constructed sequence was confirmed by Sanger sequencing performed by the Cincinnati Children’s Hospital Medical Center DNA sequencing core.

### AAV9 and MyoAAV production and in vivo injection.

AAV9 and MyoAAV vectors were produced in-house using the AAVpro 293T cell line (Takara, 632273) as described previously (35). Briefly, a 3-plasmid system is used for transfection: the pHelper plasmid (Cell Biolabs, Inc.), the AAV9 or MyoAAV capsid plasmid (36), and the promoter-driven expression plasmid containing the gene of interest. Cells at approximately 70% confluence are transfected, and the medium is replaced posttransfection with DMEM supplemented with 1% serum. After 72–96 hours, cells and media were collected for AAV purification using iodixanol gradient ultracentrifugation (15%, 25%, 40%, 60% layers) at 351,000 × *g* in a Ti70 rotor and Optiseal tubes (361625, Beckman Coulter). Iodixanol is removed by buffer exchange using Amicon Ultra-15 (UFC910024 100K, Sigma-Aldrich) and Ultra-4 filtration units (UFC803024, Sigma-Aldrich), concentrating the virus in a storage buffer (2× PBS, 5% glycerol, 1 mM MgCl_2_, 0.001% pluronic acid F-68). AAV viral DNA was extracted for qPCR-based titer calculation using a modified DNase/Proteinase K digestion protocol. Briefly, 2 μL of viral preparation was added to 100 μL DNase I solution and incubated at 37°C for 1 hour to digest free nucleic acids. DNase I was inactivated by adding 5 μL of 500 mM EDTA, followed by incubation at 75°C for 10 minutes. Next, 120 μL of Proteinase K solution was added and then incubated at 50°C for at least 2 hours or overnight to digest viral capsids and release genomic DNA. Proteinase K was subsequently inactivated by heating samples at 95°C for 10 minutes. After cooling to room temperature, 3 μL of the digested viral DNA was diluted in 897 μL of nuclease-free water (1:300 dilution). Three microliters of this diluted DNA was used as the template per qPCR. AAV titers were determined by qPCR using primers specific to the cTnT promoter region within the viral construct. The amount of viral DNA was quantified relative to a standard curve generated from a plasmid containing the same promoter sequence. Viral genome copy numbers were calculated based on the molecular weight of the full-length viral genome, including the inverted terminal repeats and gene of interest.

AAV9-*Ccl2* and control were injected intraperitoneally at 1 × 10^12^ viral genomes (vg) in 30 μL and MyoAAV-*Csf1* and control were injected via retro-orbital route at 5 × 10^10^ vg in 50 μL. Age at which virus was injected is mentioned in figure legends.

### Preparation of cardiac cell suspension.

Hearts were rapidly excised from mice, and ventricles were briefly rinsed in 1× PBS. The tissue was then minced into approximately 2 mm × 2 mm pieces using a sterile blade. Minced tissue fragments were transferred to individual wells of a 12-well tissue culture plate, each containing 2 mL of digestion buffer: DMEM containing bovine growth serum (BGS; 2%), Collagenase IV (450 U/mL LS004188, Worthington), 1.2 U/mL dispase II (D4693, Sigma-Aldrich), and 0.9 mM CaCl_2_. Plates were incubated at 37°C for 20 minutes with gentle rotation. Following incubation, the tissue was triturated manually 15–20 times with a 10 mL serological pipette until the pieces could pass through the pipette freely. Tissue pieces were allowed to settle by gravity, and the supernatant was collected and passed through a prewet 40 μm cell strainer (22363547, Thermo Fisher Scientific) into a 50 mL Falcon tube (Corning) placed on ice. A fresh 2 mL of digestion buffer was added, and the digestion cycle was repeated twice by triturating tissue pieces with 5 mL serological pipette for round 2 and with 1 mL pipette for round 3 for improved dissociation. The supernatant was collected from each digestion round and pooled passing through same cell strainer. Cells were collected by centrifugation at 250*g* for 15 minutes at 4°C. The resulting supernatant was discarded, and the pellet was resuspended in 1 mL of RBC lysis buffer (R7757, Sigma-Aldrich) for ~90 seconds to remove erythrocyte contamination. RBC lysis conditions were neutralized by adding 10 mL of 1× HBSS containing 2% BGS, followed by another centrifugation at 250*g* for 15 minutes at 4°C. The final cell pellet was resuspended in FACS buffer (1× HBSS, 2% BGS, and 2 mM EDTA) and processed for downstream flow cytometry staining as described previously (37).

### Flow cytometry and gating strategy.

Cardiac single-cell suspensions were prepared as described above and resuspended in 500 μL of FACS buffer (1× HBSS, 2% BGS, and 2 mM EDTA). Cells were stained with fluorophore-conjugated monoclonal antibodies for 25 minutes at 4°C in the dark. Samples were washed twice with 1 mL of FACS buffer and filtered through 35 μm mesh cell strainer tubes (Falcon, Corning, 352235). Flow cytometry was performed using an LSR Fortessa (BD Biosciences) running BD Biosciences FACSDiva, and data were analyzed with FlowJo software v10 (BD Biosciences). Single cells were identified and gated based on forward scatter and side scatter. Compensation was performed for each experiment using unstained and single-stained control samples prepared from cardiac cell suspensions of wild-type mouse hearts as described previously (34). Cardiac macrophages were gated as CD45^+^CD11b^+^CD64^+^, and then CX3CR1^+^ or CCR2^+^ subtypes were gated based on endogenous GFP or RFP expression, respectively. MHC-II or TIMD4 markers were used to further classify their subpopulations. *Cx3cr1* lineage-derived cardiac macrophages were gated as CCR2^–^tdTom^+^. Neutrophils were gated as CD45^+^CD11b^+^Ly6G^+^, total T lymphocytes were gated as CD45^+^CD11b^–^NK1.1^–^CD3^+^, CD4^+^ T cells were gated as CD45^+^CD11b^–^NK1.1^–^CD3^+^CD4^+^, and CD8^+^ T cells were gated as CD45^+^CD11b^–^NK1.1^–^CD3^+^CD8^+^. B lymphocytes were gated as CD45^+^CD11b^–^NK1.1^–^CD3^–^CD19^+^. NK cells were gated as CD45^+^CD11b^–^CD3^–^NK1.1^+^. Cardiac fibroblasts were gated as CD45^–^CD31^–^MEFSK4^+^. The following mouse antibodies were used: CD45 (30-F11), CD11b (M1/70), CD64 (X54-5/7.1), Ly6G (1A8), CD11c (N418), Ly6C (HK1.4), MHC-II (AF6-120.1), TIMD4 (RMT4-54), CD3 (17A2), CD19 (6D5), NK1.1 (PK136), CD31 (390), CCR2 (475301), and Anti-feeder cells (MEFSK4). All antibodies were purchased from BioLegend except Ly6G (BD Biosciences), CCR2 (R&D Systems), and Anti-feeder cells (Miltenyi Biotec). All flow cytometry antibodies were used at 1:200 dilution except CCR2 (1:50) and Anti-feeder cells (1:50). Flow cytometry antibody details are provided in Supplemental Table 1.

### Histology and immunofluorescence.

Hearts and other tissues were harvested and fixed overnight in 4% paraformaldehyde in 1× PBS at 4°C with gentle agitation. Following fixation, tissues were washed twice with 10 mL of cold 1× PBS and cryopreserved in 30% sucrose (w/v) for at least 24 hours. Tissues were then embedded in Optimal Cutting Temperature (OCT; Tissue-Tek O.C.T. Compound, 4583, SAKURA) and flash-frozen in 2-methylbutane chilled with dry ice as described by us previously (37). Frozen blocks were sectioned at 8 μm thickness using a cryomicrotome (Leica CM1860). All histological stainings were performed on OCT-embedded frozen sections. For immunostaining, frozen sections were brought to room temperature and washed twice with 1× PBS to remove residual OCT. Sections were then blocked in 5% normal donkey serum (017-000-121, Jackson ImmunoResearch) for 1 hour at room temperature and incubated overnight at 4°C with primary antibodies: anti-GFP (1:200), anti-RFP (1:200), anti-CD68 (1:200), anti-PDGFRα (1:200), anti-POSTN (1:100), and anti-vimentin (1:100). After incubation, sections were washed 3 times with 1× PBS and incubated with fluorochrome-conjugated appropriate secondary antibodies (1:400; Jackson ImmunoResearch) for 1 hour at room temperature. Following secondary antibody incubation, sections were washed 3 times with 1× PBS. Nuclei were stained with DAPI (1:10,000, D3571, Invitrogen), and Alexa Fluor 647–conjugated WGA (1:200, W32466, Invitrogen) was used for membrane staining. Sections were washed 3 times with 1× PBS and mounted with ProLong Diamond Antifade (P36961, Thermo Fisher Scientific) and coverslips (1415-10, Globe Scientific). Secondary antibody–only controls were included in all immunofluorescence experiments to confirm staining specificity. Confocal imaging was performed using a Nikon Eclipse Ti inverted microscope equipped with a Nikon A1R confocal system operated with NIS-Elements software. Representative images were processed using the same software. Immunofluorescence antibody details are provided in Supplemental Table 2.

### Fibrosis quantification.

Masson’s trichrome staining was performed on 8 μm frozen heart sections at the Pathology Core at Cincinnati Children’s Hospital Medical Center. Each mouse heart was bisected, and a minimum of 4 sections were prepared from each half to ensure coverage across a reasonable tissue span. Images were acquired using M165 FC stereomicroscope (Leica Microsystems). The percentage of blue-stained area (indicating fibrosis) relative to the total tissue area was quantified using ImageJ software (38) (NIH) as we described previously (39).

### ELISA.

Levels of CCL2 protein in both cardiac tissue and serum were measured using a commercially available ELISA kit (R&D Systems; MJE00B), following the manufacturer’s protocol. Similarly, cardiac tissue and serum levels of CSF1 were quantified using a separate ELISA kit (R&D Systems; MMC00B), also according to the manufacturer’s protocol. Heart tissue was undetectable at baseline in MyoAAV-Empty–infected mice under our assay conditions, so a value of 0 was used in Figure 4B. Absorbance of the ELISA kit readings was measured using a microplate reader (BioTek Instruments), and concentrations were calculated based on standard curves generated with known concentrations of recombinant proteins provided in the kit.

### RNA isolation, qRT-PCR, and RNA sequencing.

Total RNA from cardiac tissue was isolated using the RNeasy Kit (QIAGEN) following the manufacturer’s protocol. We reverse-transcribed 1 μg of RNA using Superscript III Reverse transcriptase (18080044, Thermo Fisher Scientific), Random hexamers (48190011, Thermo Fisher Scientific), dNTP blend (N8080260, Thermo Fisher Scientific), and Ribonuclease inhibitor (10-777-019, Thermo Fisher Scientific). qRT-PCR was performed using the CFX384 Touch Real-Time PCR detection system (Bio-Rad) and SYBR Select Master Mix (4472942, Thermo Fisher Scientific) as described previously (40). Target gene expression levels were normalized to either mouse hypoxanthine guanine phosphoribosyl transferase or mouse 18S ribosomal RNA, used as endogenous reference genes. The ΔΔCq (Cq refers to quantification cycle) method was applied to quantify fold-change in expression relative to a designated control sample. qRT-PCR primer sequences were provided in Supplemental Table 3. For bulk RNA sequencing, total RNA from cardiac tissue was isolated and shipped to Novogene (California, USA) for quality assessment and sequencing (Supplemental Figure 6D data). Briefly, mRNA was purified from total RNA using poly-T oligo-attached magnetic beads. After fragmentation, the first-strand cDNA was synthesized using random hexamer primers, followed by the second-strand cDNA synthesis for library generation. The library was checked with Qubit and real-time PCR for quantification and Agilent Bioanalyzer for size distribution detection. Quantified libraries were pooled and sequenced using Illumina NovaSeq X-Plus Sequencing Platform (read length: 150 bp, paired-end). Differential gene expression analysis between experimental groups was performed using the DESeq2 R package (1.42.0); the resulting *P* value was adjusted using Benjamini-Hochberg methods to control the error discovery rate. For bulk RNA-sequencing data in Supplemental Figure 7F, sequencing was performed by Plasmidsaurus Inc. using an Illumina-based 3′ end counting workflow (41). Raw reads were processed for adapter and quality trimming, followed by alignment to the reference genome using STAR. Unique molecular identifier–based deduplication was applied prior to downstream analysis. Gene-level counts were generated using FeatureCounts with strand-specific assignment and annotated using the reference GTF. Quality control metrics were assessed across alignment and feature distributions. Differential expression analysis was performed using edgeR with standard low-expression filtering.

### TAC and echocardiography.

Mice were anesthetized with 3% isoflurane before intubating using an 18-gauge catheter and were ventilated throughout the surgical procedure using a SomnoSuite system (TSE Systems) and then given 1.7% isoflurane. A thoracotomy was performed to expose and isolate the transverse aorta as we have shown previously (42). To induce constriction, a suture was tied around the transverse aorta alongside a 26-gauge, blunt-end needle, which was then removed. The thoracic incision was sutured and further sealed with GLUture (Zoetis, Butler Schein, 034418). Following extubation, mice received a subcutaneous injection of sustained-release buprenorphine (3.25 mg/kg body weight) for pain management. Postoperative monitoring was conducted daily. Sham surgeries followed the same procedure, except that no suture was placed around the transverse aorta. For echocardiographic analysis of cardiac structure and function, mice were anesthetized with 2% isoflurane in a 100% oxygen mix. Mice were secured to a temperature-controlled board equipped with electrocardiogram sensors (Fujifilm VisualSonics), and heart rate was monitored throughout imaging. Echocardiography was performed with a Vevo 3100 (Fujifilm VisualSonics) using an MS550D probe and analyzed by Vevo Lab software (Fujifilm VisualSonics).

### Hemodynamic measurement by catheterization.

In vivo cardiac hemodynamics were measured in mice anesthetized with 1.75% isoflurane using a pressure-volume catheter (1.2F, Transonic System Inc) inserted into the left ventricle via cannulation of the right carotid artery as we have described previously (43). Data were collected on PowerLab LabChart 8 (ADInstruments).

### Single-nucleus sequencing and analysis.

Mouse hearts were flash-frozen in liquid nitrogen and stored at –80°C until processing. Frozen tissue was crushed into a powder in liquid nitrogen using a mortar and pestle, then resuspended in 200 μL Nuclei EZ lysis buffer (Sigma, NUC-101) containing 1 U/mL RNase inhibitor and 0.5% BSA. Samples were homogenized using a Dounce homogenizer (Pestle A, 20–22 strokes), incubated on ice for 10 minutes, and sequentially filtered through 100, 40, and 20 μm strainers (22-363-549 and 22-363-547, Thermo Fisher Scientific). The flow-through was centrifuged at 500*g* for 6 minutes at 4°C. The pellet was gently resuspended in 1× PBS with 2% BSA and 0.5 U/mL RNase inhibitor, incubated on ice for 5 minutes, and centrifuged at 500*g* for 5 minutes at 4°C. The resulting pellet was resuspended in 1× PBS with 1% BSA, 0.5 U/mL RNase inhibitor, and DAPI (1:1,000; 5 mg/mL), then incubated on ice for 10 minutes. After centrifugation at 500*g* for 5 minutes at 4°C, the supernatant was discarded. The final pellet was resuspended in 1× PBS with 1% BSA and 1 U/mL RNase inhibitor. DAPI^+^ nuclei were isolated via flow cytometry sorting using BD Biosciences FACSAria II system. Sorted nuclei were filtered through a 20 μm strainer and centrifuged at 1,000*g* for 5 minutes at 4°C. The final pellet was resuspended in 1× PBS with 1% BSA and 1 U/mL RNase inhibitor at 500–1,000 nuclei/mL and submitted for nuclear integrity assessment and 10x Genomics nuclear capture. The 10x Chromium platform was used to load the nuclei according to the Single Cell 3′ Reagent Kit v3.1, following manufacturer’s guidelines. Sequencing was performed on an Illumina NovaSeq 6000 System at the Cincinnati Children’s Hospital Medical Center, Division of Human Genetics. 10x Genomics single-nucleus RNA sequencing FASTQs were processed with Cell Ranger (44) (cellranger count) to generate gene-by-barcode matrices. Ambient/background RNA was removed per sample with CellBender (remove-background), producing corrected H5 matrices. CellBender outputs were loaded into R/Seurat to create an integrated object (seurat_integrated.rds). Downstream analyses used the RNA assay. Preprocessing and clustering were performed separately for the whole dataset and cardiac macrophage subsets using the following workflow: NormalizeData, FindVariableFeatures, ScaleData, RunPCA, FindNeighbors, and FindClusters. Low-dimensional embeddings were computed with UMAP [RunUMAP(dims = 1:15)] and t-SNE [RunTSNE(dims = 1:15)] using the uwot and Rtsne back ends, respectively.

### Statistics.

All data are presented as mean ± SEM. GraphPad Prism 9 software was used to perform all statistical analyses. An unpaired 2-tailed Student’s *t* test was used to compare 2 experimental groups, and 1-way ANOVA with Tukey’s multiple comparisons test was used to compare more than 2 experimental groups. For all tests, *P* < 0.05 was considered significant.

### Study approval.

All experimental procedures with animals were approved by the Institutional Animal Care and Use Committee of Cincinnati Children’s Medical Center, protocol IACUC 2025-0047. We have complied with the relevant ethical considerations for animal usage overseen by this committee as described in the *Animals* section of the Methods.

### Data availability.

The bulk RNA-sequencing data related to Supplemental Figure 6D are available at the National Center for Biotechnology Information Gene Expression Omnibus (accession number GSE307501) and related to Supplemental Figure 7F are available at the Gene Expression Omnibus (accession number GSE326299). The single-nucleus RNA-sequencing dataset is accession number GSE307775. Source data underlying the graphs in the main and supplemental figures are provided in the Supporting Data Values file.

## Author contributions

RKK and JDM conceptualized the study. RKK, RJV, YK, AKZJ, NSB, VP, SCJL, AR, and MN completed experiments and analyzed data. RKK and JDM wrote the manuscript, and all other authors provided comments. JDM acquired funding and supervised all experimentation.

## Conflict of interest

The authors have declared that no conflict of interest exists.

## Funding support

This work is the result of NIH funding, in whole or in part, and is subject to the NIH Public Access Policy. Through acceptance of this federal funding, the NIH has been given a right to make the work publicly available in PubMed Central.

- NIH (1R01HL156852, 1R01HL160765, 1P01HL160488, to JDM).
- American Heart Association (Postdoctoral Fellowship 903556, to RKK, and Career Development Award 24CDA1274099, to YK).

## Supplementary Material

Supplemental data

Supporting data values

## Version 1 06/09/2026

In-Press Preview

## Version 2 08/03/2026

Electronic publication
