# Supplementary material for: Selective expansion of cardiac macrophage subtypes distinguishes their functional roles in disease and homeostasis
Source: J Clin Invest. 2026 Jun 9;136(15):e200194. doi: 10.1172/JCI200194 (PMC13430008; doi:10.1172/JCI200194)
Supplement: Supplemental data [file jci-136-200194-s151.pdf]

Selective expansion of cardiac macrophage subtypes distinguishes their functional roles in disease and homeostasis

Rajesh K. Kasam[1], Ronald J. Vagnozzi[2], Yasuhide Kuwabara[1], Anne Katrine Z Johansen[1], N. Scott Blair[1], Vikram Prasad[1], Suh-Chin J. Lin[1], Akanksha Rajput[1], Michelle Nieman[1], Jeffery D. Molkentin[1]

[1]Department of Pediatrics, University of Cincinnati, Cincinnati Children's Hospital Medical Center, Cincinnati, OH, USA.

[2]Division of Cardiology, Department of Medicine, University of Colorado Anschutz Medical Campus, Aurora, CO, USA.

Supplementary Figures 1 – 10

Supplementary Tables 1 – 3

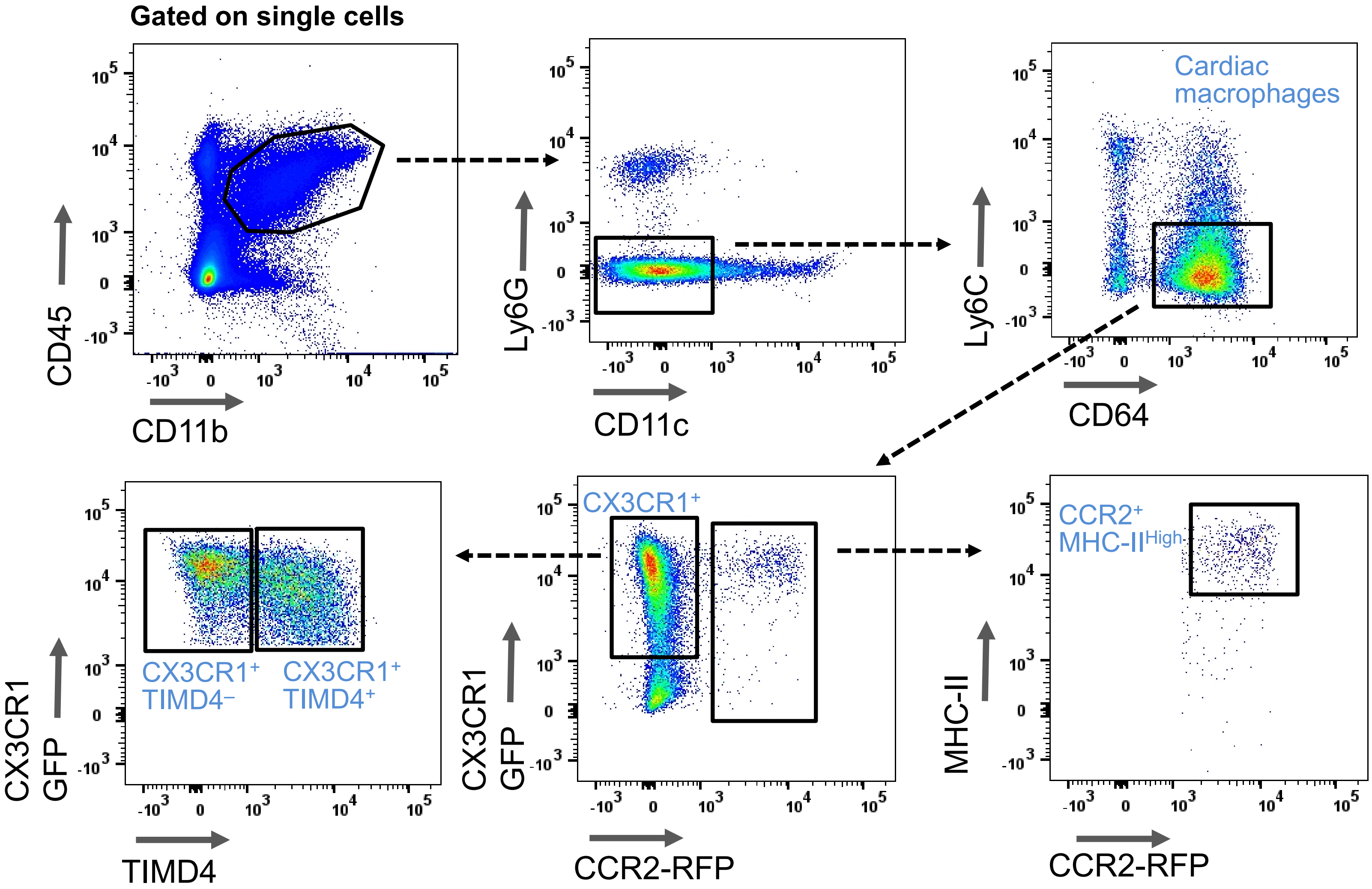

Supplementary Figure 1: Flow cytometry gating strategy used for total cardiac macrophages and their subtype identification in 2 months, 7 months, 15 months and 24 months old mice (**Figure 1** data). Neutrophils (Ly6G$^+$), dendritic cells (CD11c$^+$) and monocytes (Ly6C$^+$) were excluded, and cardiac macrophages were identified based on CD45, CD11b and CD64 expression, followed by their subtype identification; CCR2$^+$ MHC-II^{High}, CX3CR1$^+$ TIMD4$^+$, and CX3CR1$^+$ TIMD4$^-$. CCR2 and CX3CR1 identification was performed based on endogenous RFP or GFP expression from respective alleles.

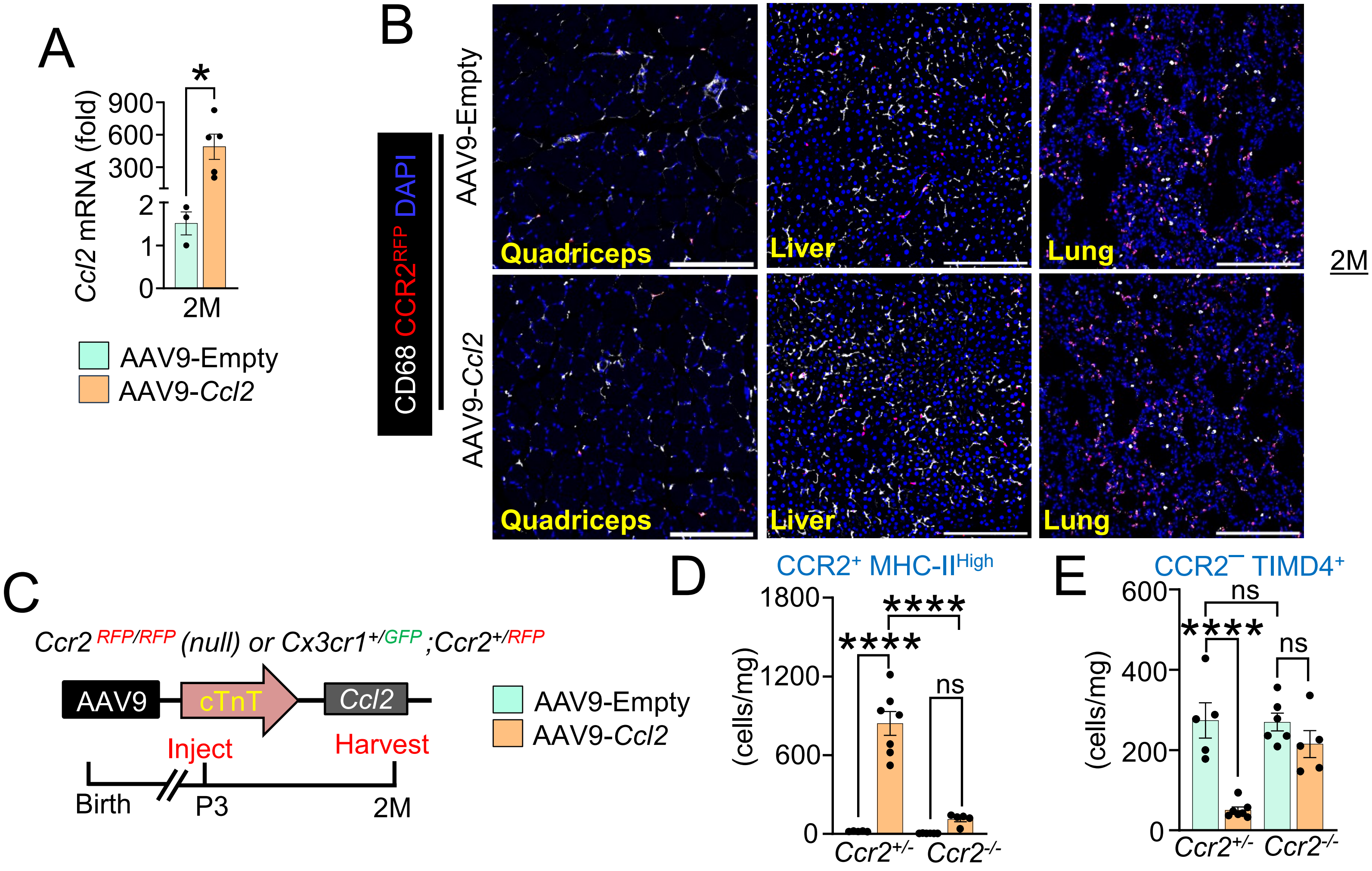

Supplementary Figure 2: (**A**) Quantitative real-time PCR (qRT-PCR) analysis of *Ccl2* gene expression in cardiac tissue of AAV9-Empty or AAV9-*Ccl2* injected mice injected at P3 and harvested at 2 months of age. n=3-5 mice per group and, error bars denote ± SEM, *P<0.05, by two-tailed unpaired Student's t test. (**B**) Representative immunofluorescence histological images in the three indicated tissues from the two groups of mice injected at P3 and then harvested at 2 months of age stained with the macrophage marker CD68 (white), RFP (CCR2, red) and nuclei (DAPI, blue). n=4 mice per group. Scale bar = 200 µm. (**C**) Experimental scheme showing AAV9-Empty or AAV9-*Ccl2* vector delivery in 3-day (P3) old pups of the genotype *Cx3cr1*$^{+/GFP}$*; Ccr2*$^{+/RFP}$ (reporter mice) or *Ccr2*$^{RFP/RFP}$ (*Ccr2* null mice) and tissue harvested at 2 months of age. (**D, E**) Quantification of CCR2^{+} MHC-II^{High} (**D**), and CCR2$^{–}$ TIMD4^{+} (**E**) cardiac macrophages from hearts of both groups of mice of the indicated genotype injected at P3 and harvested at 2 months of age. n=5-7 mice per group and, error bars denote ± SEM, ****P <0.0001 by One-way ANOVA with Tukey's multiple comparison test. ns, non-significant.

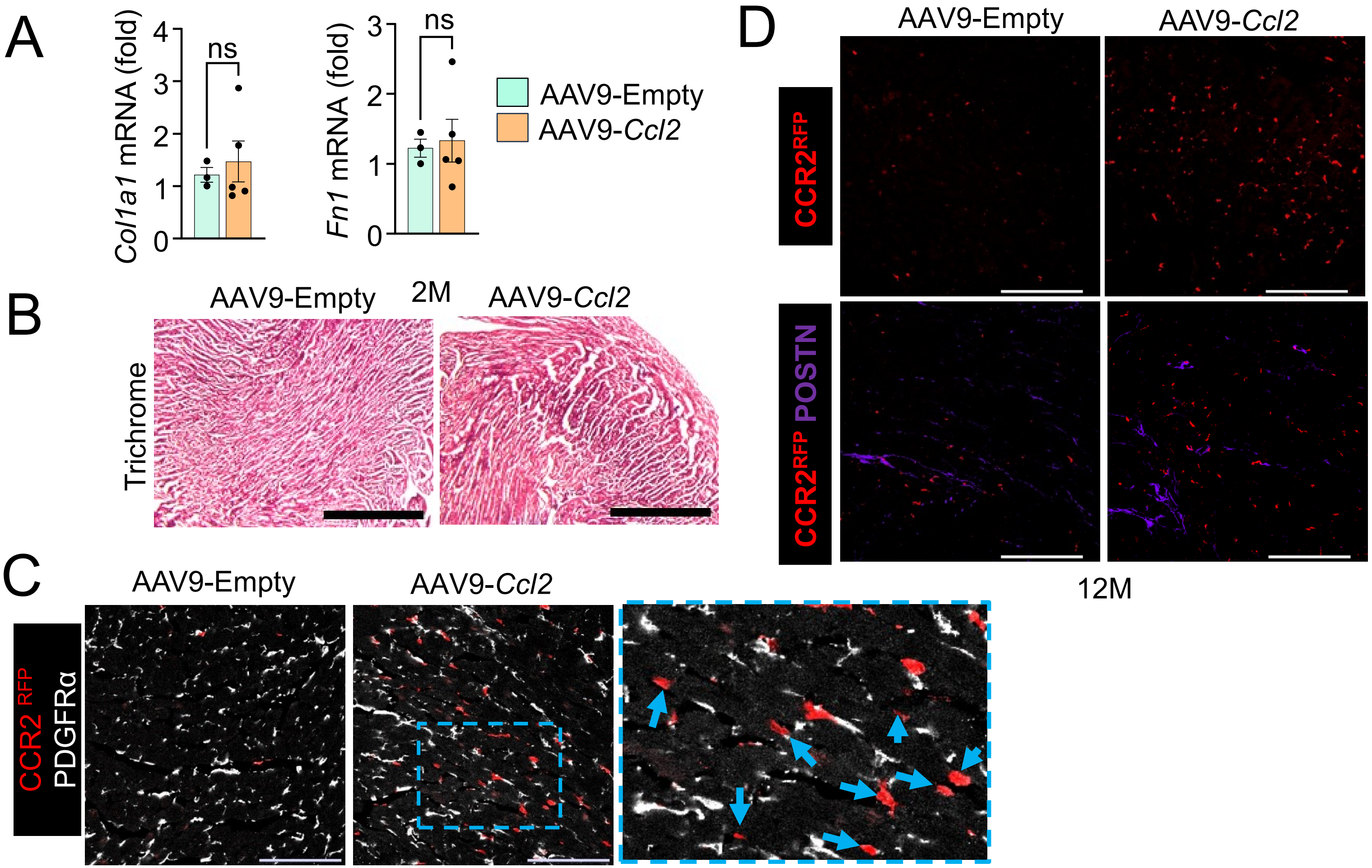

Supplementary Figure 3: (**A**) qRT-PCR analysis of *Col1a1* and *Fn1* gene expression in cardiac tissue of AAV9-Empty or AAV9-*Ccl2* injected mouse pups at P3 and harvested at 2 months of age. n=3-5 mice per group and, error bars denote ± SEM by two-tailed unpaired Student's t test. ns, non-significant. (**B**) Representative Masson's trichrome stained cardiac histological images from the two indicated groups of mice harvested at 2 months of age. Scale bar = 200 µm. (**C**) Representative immunofluorescent cardiac histological images from the two indicated groups of mice stained for RFP in red ($CCR2^+$) and PDGFRα in white (fibroblasts) at 2 months of age with prior injection of AAV9-*Ccl2* in P3 pups. The boxed area on the image from the AAV9-*Ccl2* treated mice was blown up on the right and the arrows show $CCR2^+$ macrophages that are not enriched for interaction with fibroblasts. However, **Supplementary Figure 8** shows that $CX3CR1^+$ tissue resident macrophages are enriched for direct association with cardiac fibroblasts in the heart. n=4 mice per group. Scale bar = 200 µm. (**D**) Representative immunofluorescence cardiac histological images stained for RFP in red ($CCR2^+$) and periostin in purple (POSTN) from AAV9-Empty or AAV9-*Ccl2* injected into P3 mouse pups and subsequently harvested at 12 months of age. Scale bar = 200 µm. n=4 mice per group.

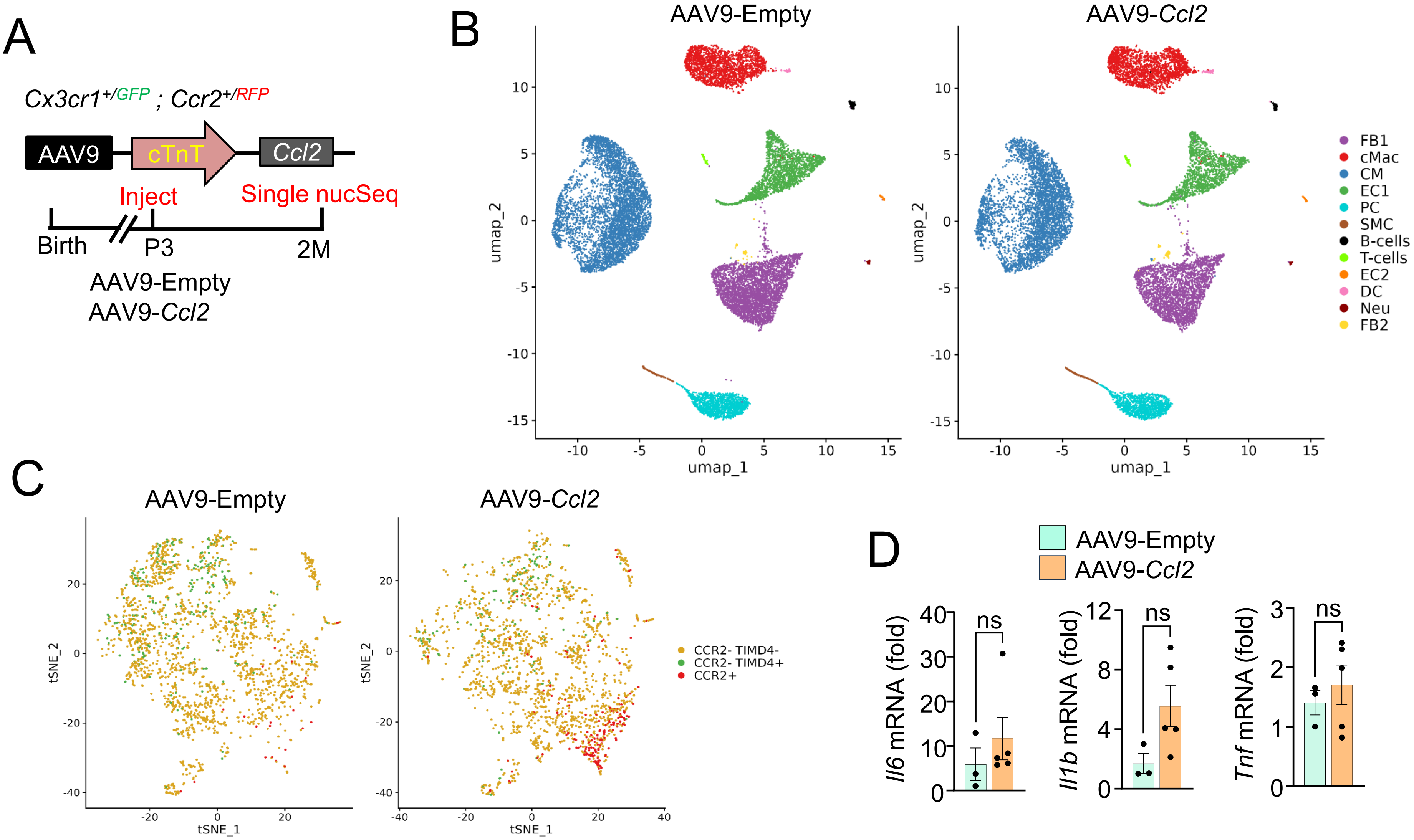

Supplementary Figure 4: (A) Experimental scheme showing AAV9-*Ccl2* recombinant vector driven by the cTnT promoter, which was injected in the indicated reporter mice at P3 and then the hearts were harvested at 2 months of age for cardiac single nucleus sequencing. n=2-3 mice per group. (**B**) Uniform manifold approximation and projection (UMAP) of single nucleus sequencing data from hearts of AAV9-Empty and AAV9-*Ccl2* groups. Legend; FB1 (fibroblasts cluster 1), cMac (cardiac macrophages), CM (cardiomyocytes), EC1 (endothelial cell cluster 1), PC (pericytes), SMC (smooth muscle cells), EC2 (endothelial cell cluster 2), DC (dendritic cells), Neu (neuronal cells), FB2 (fibroblast cluster 2). (**C**) t-distributed stochastic neighbor embedding (tSNE) plot depicting 3 major cardiac macrophage subtypes in the heart from the data in B, showing increased CCR2^{+} macrophage content by mRNA for Ccr2 and/or Timd4 due to *Ccl2* overexpression. The data show increased CCR2^{+} expressing cells and reduced TIMD4^{+}. (**D**) qRT PCR gene expression analysis from the two groups of mice from cardiac tissue for *Il6*, *Il1b* and *Tnf* genes at 2 months of age. n=3-5 mice per group and, error bars denote ± SEM by two-tailed unpaired Student's t test. ns, non-significant.

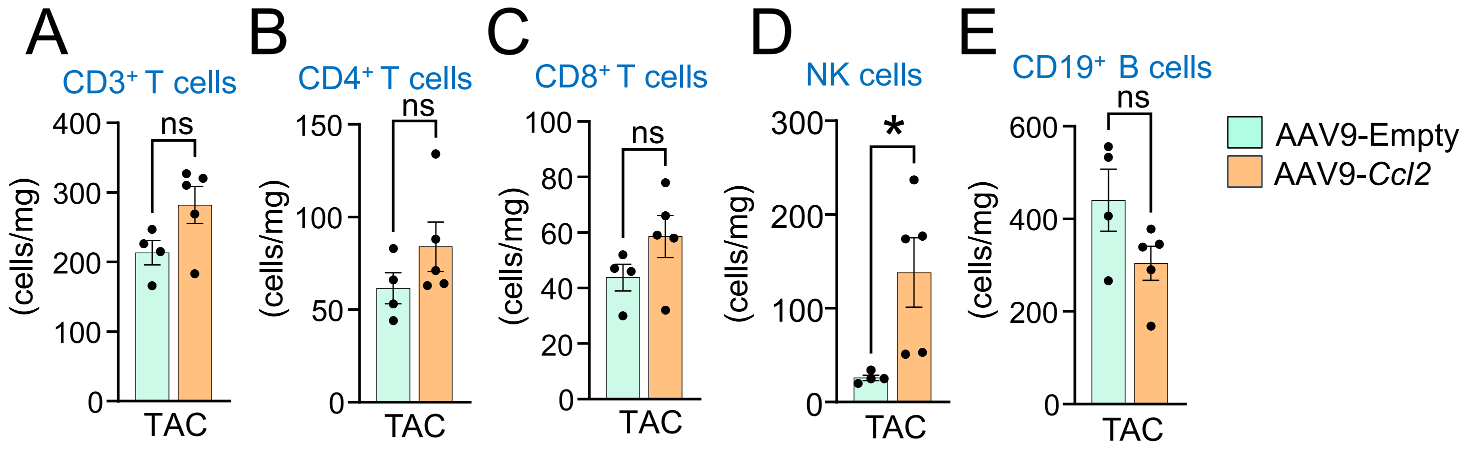

Supplementary Figure 5: AAV9-Empty or AAV9-*Ccl2* was delivered in adult reporter mice and subjected to TAC as shown in **Figure 3A**. (**A-E**) Flow cytometry quantification of indicated cell types in hearts of mice with AAV9-Empty and AAV9-*Ccl2* injection and then harvested 8 weeks after TAC as (**A**) $CD3^+$ total T-cell, (**B**) $CD4^+$ T cells, (**C**) $CD8^+$ T cells, (**D**) NK cells, and (**E**) $CD19^+$ B cells. n=4-5 mice per group, and error bars denote ± SEM, *P<0.05, by two-tailed unpaired Student's t test. ns, non-significant.

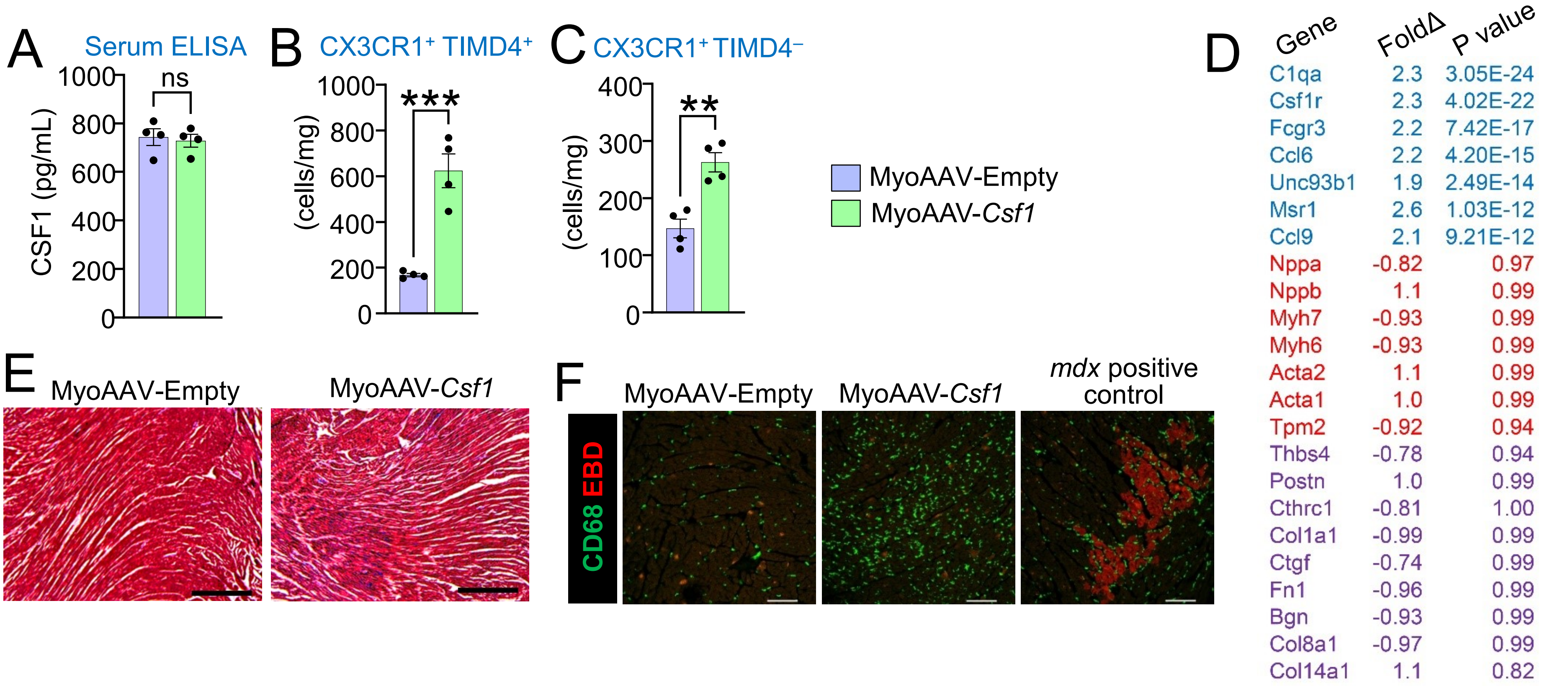

Supplementary Figure 6: (A) Quantification of serum CSF1 levels by ELISA in adult reporter mice injected with MyoAAV-Empty or MyoAAV-*Csf1* and harvested 3 weeks later. n=4 mice per group, and error bars denote ± SEM, ns, non-significant, by two-tailed unpaired Student's t test. (**B-C**) Flow cytometry quantification from hearts of the indicated macrophage subtypes in MyoAAV-Empty and MyoAAV-*Csf1* injected mice 3 weeks later as (**B**) CX3CR1^{+} TIMD4^{+}, (**C**) CX3CR1^{+} TIMD4^{-}. n=4 mice per group, and error bars denote ± SEM, **P<0.01, ***P <0.001, by two-tailed unpaired Student's t test. (**D**) Selected genes from RNA sequencing analysis as fold change and p-values from hearts of adult mice 6 weeks after MyoAAV-Empty or MyoAAV-*Csf1* injection. n=4 mice group. The global gene expression analysis showed no induction of cardiac stress markers or fetal genes (red text) due to 6 weeks of *Csf1* overexpression, nor activation of fibrotic genes (purple text), although there was a significant induction of select inflammatory like genes (blue text). (**E**) Representative cardiac histology stained with Masson's trichrome (fibrosis would be blue) from the 2 indicated groups of mice that were harvested 16 weeks after control or MyoAAV-*Csf1* injection. n=4-5 mice per group. (**F**) Representative immunofluorescence cardiac histological images for Evan blue dye (EBD; red) uptake as an indication of necrosis or membrane leakiness and CD68 (green) to show macrophages in WT adult mice injected 6 weeks earlier with MyoAAV-Empty or MyoAAV-*Csf1*. EBD was injected 24 hrs prior to tissue harvest. Cardiac tissue from muscular dystrophy mice (*mdx*) was used as a positive control for EBD uptake with membrane leakiness. n=5 mice per group. Scale bar = 100 μm.

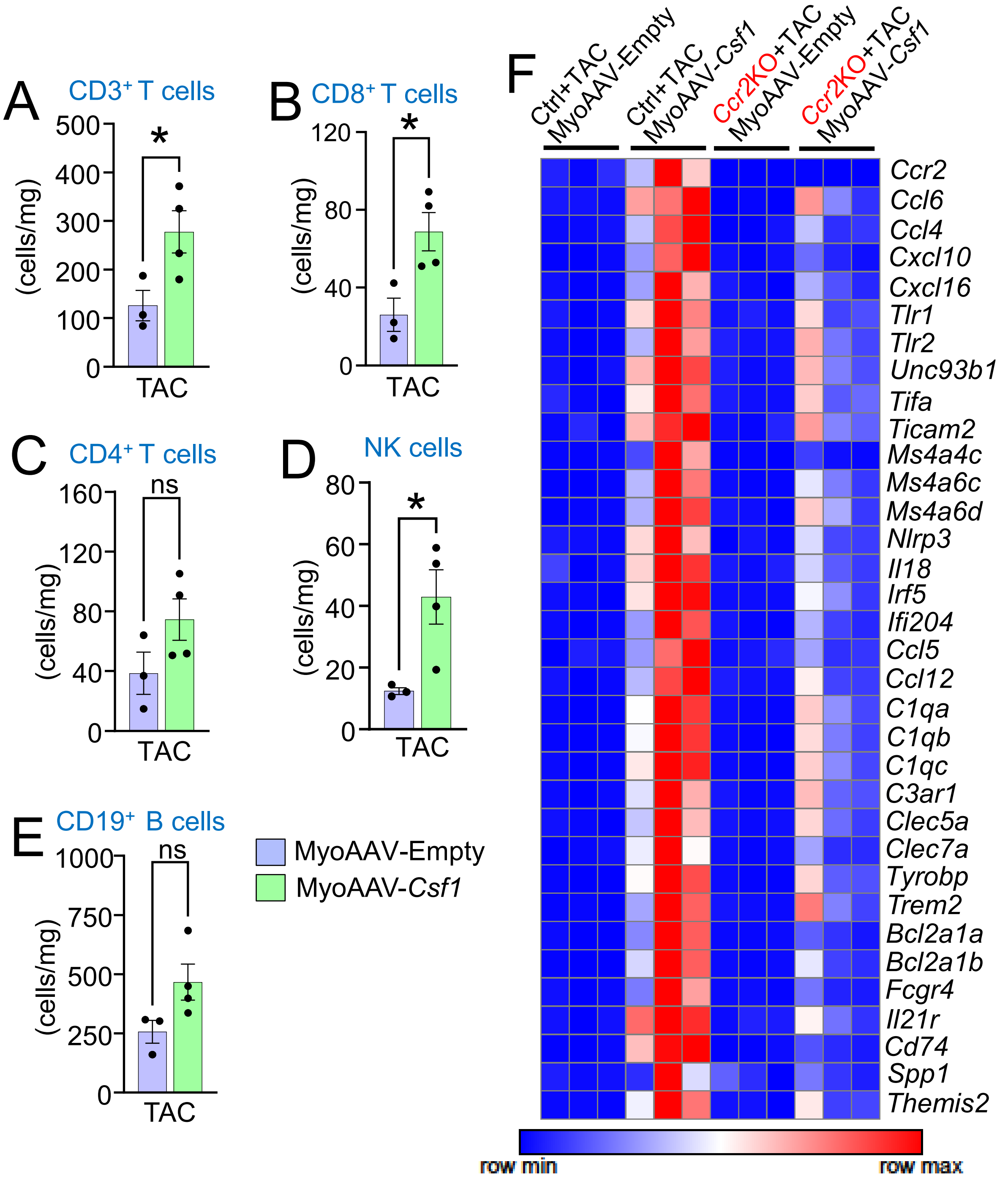

Supplementary Figure 7: MyoAAV-Empty or MyoAAV-*Csf1* vector was injected in adult reporter mice at 8 weeks of age and subjected to TAC or sham surgery and harvested 3 weeks later with harvesting 8 weeks later as shown in **Figure 6A** (19 weeks of age). (**A-E**) Flow cytometry quantification of indicated cell types in heart tissue of these mice as (**A**) $CD3^+$ total T-cells, (**B**) $CD8^+$ T cells, (**C**) $CD4^+$ T cells, (**D**) NK cells, and (**E**) $CD19^+$ B cells. n=3-4 mice per group, and error bars denote ± SEM, $^*P<0.05$, by two-tailed unpaired Student's t test. ns, non-significant. (**F**) Heatmap showing selected pro-inflammatory genes from bulk-RNA sequencing analysis performed in LV heart samples from reporter mice (Ctrl mice) or *Ccr2* null mice (KO) from the indicated viral vectors and subjected to TAC and harvested 8 weeks later as shown in **Figure 6A** and **Figure 7A**. n=3 mice per group. The data show induction of inflammation with *Csf1* overexpression after TAC in control mice (**Figure 6**) but much less so in *Ccr2* null mice (KO) with *Csf1* overexpression and TAC (**Figure 7**).

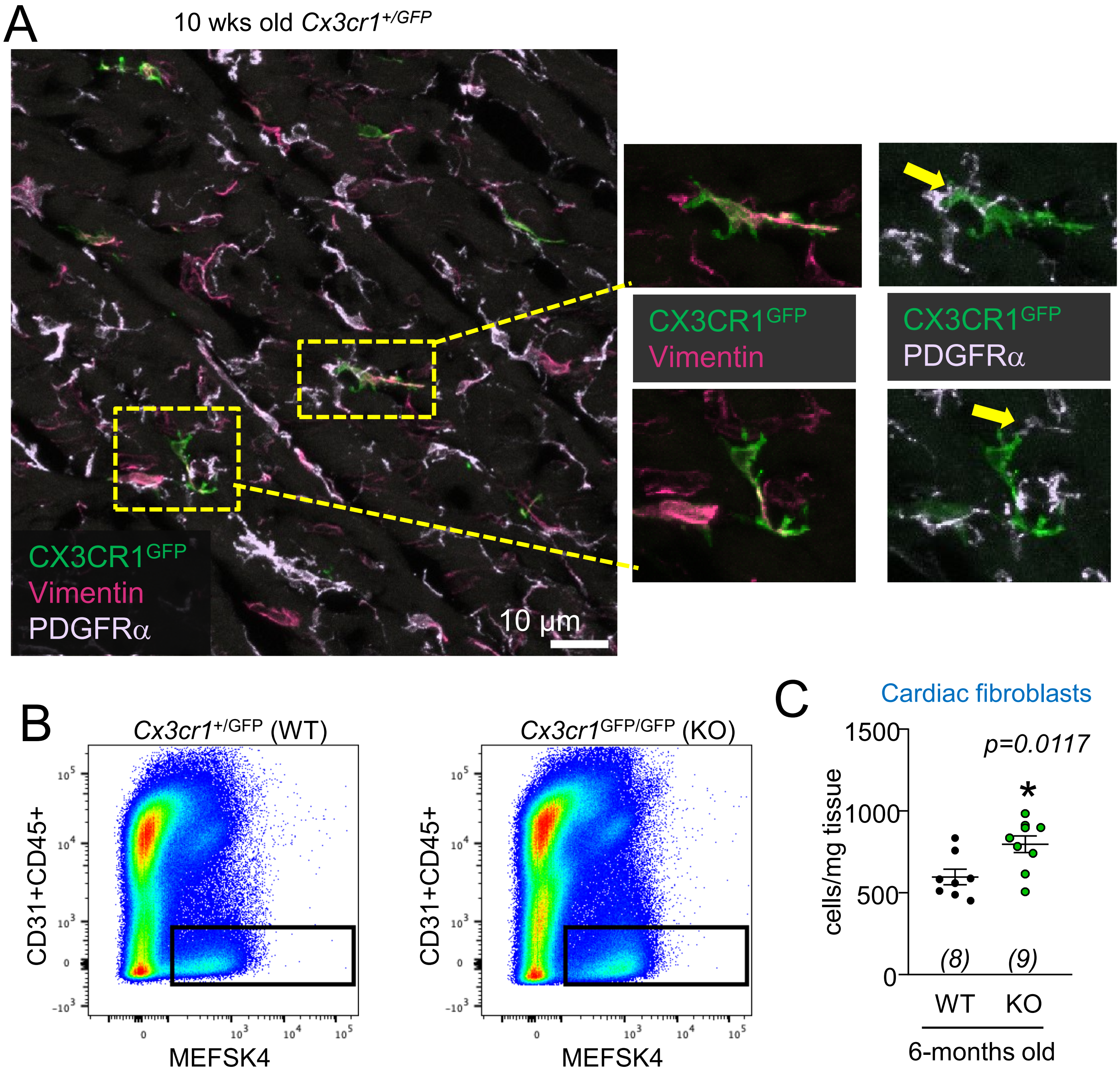

Supplementary Figure 8: (**A**) Representative immunofluorescence images from cardiac histological sections from 10 wk old Cx3cr1$^{+/GFP}$ reporter mice stained with vimentin (pink), PDGFRα (white) for fibroblasts and GFP to mark CX3CR1^{+} cells (green). Highlighted boxed areas shows CX3CR1^{GFP} macrophages in direct association with vimentin and PDGFRα fibroblasts. Scale bar = 10 μm. (**B, C**) Flow cytometry plots showing cardiac fibroblasts gated as CD45^{-}CD31^{-}MEFSK4^{+} cells (**B**), and their quantification (**C**), from hearts of 6 months old *Cx3cr1*$^{+/GFP}$ (WT) and *Cx3cr1*$^{GFP/GFP}$ (KO) mice. n=8-9 mice per group and, error bars denote ± SEM, *P<0.05, by Student's t-test.

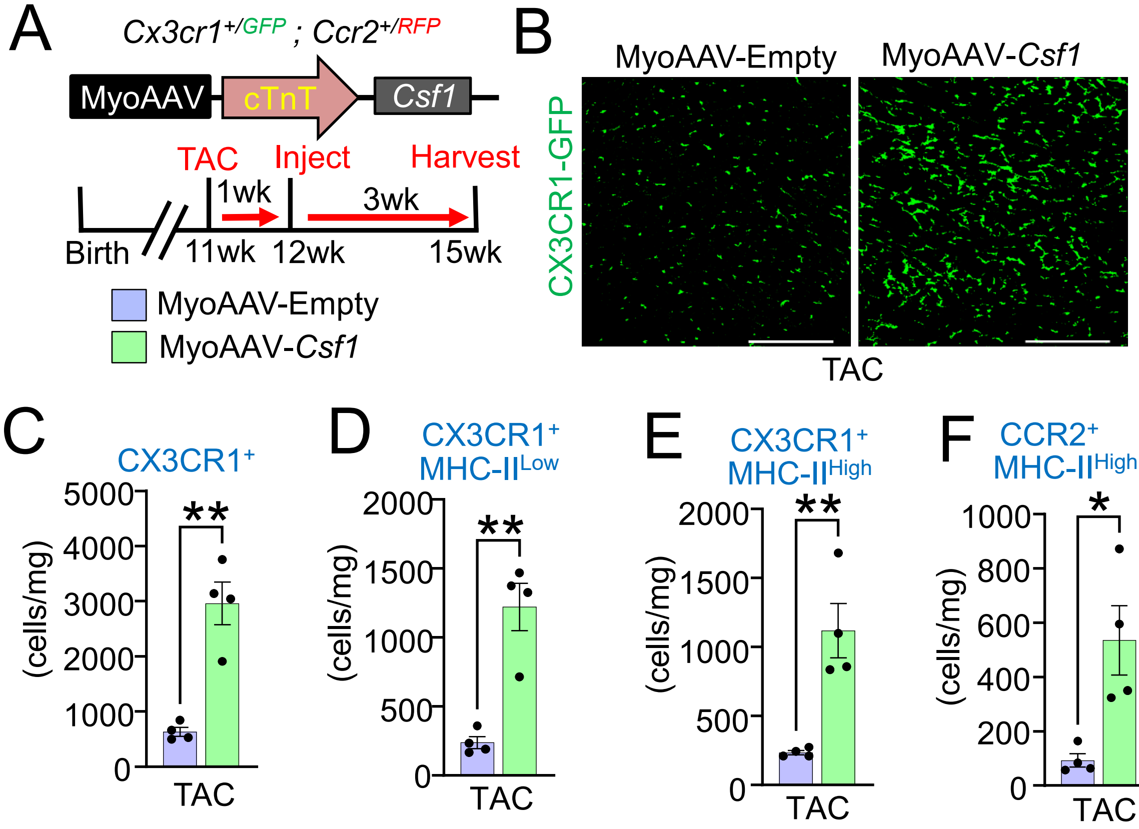

Supplementary Figure 9: (**A**) Experimental scheme showing adult reporter mice subjected to TAC at 11 weeks of age and injected with either MyoAAV-Empty or MyoAAV-*Csf1* vector 1 week after TAC and harvested 3 weeks later (4 weeks of TAC). (**B)** Representative immunofluorescence images from heart tissue sections of GFP (CX3CR1$^+$) cells in the two groups of mice 4 weeks after TAC. n=4 mice per group, scale bar = 200 μm. (**C-F**) Flow cytometry quantitation of macrophage subtypes in hearts of these two groups of mice as (**C**) total CX3CR1$^+$, (**D**) CX3CR1$^+$ MHC-II^{Low}, (**E**) CX3CR1$^+$ MHC-II^{High}, and (**F**) CCR2$^+$ MHC-II^{High} macrophages. n=4 mice per group, and error bars denote ± SEM, *P<0.05, **P<0.01, by two-tailed unpaired Student's t test.

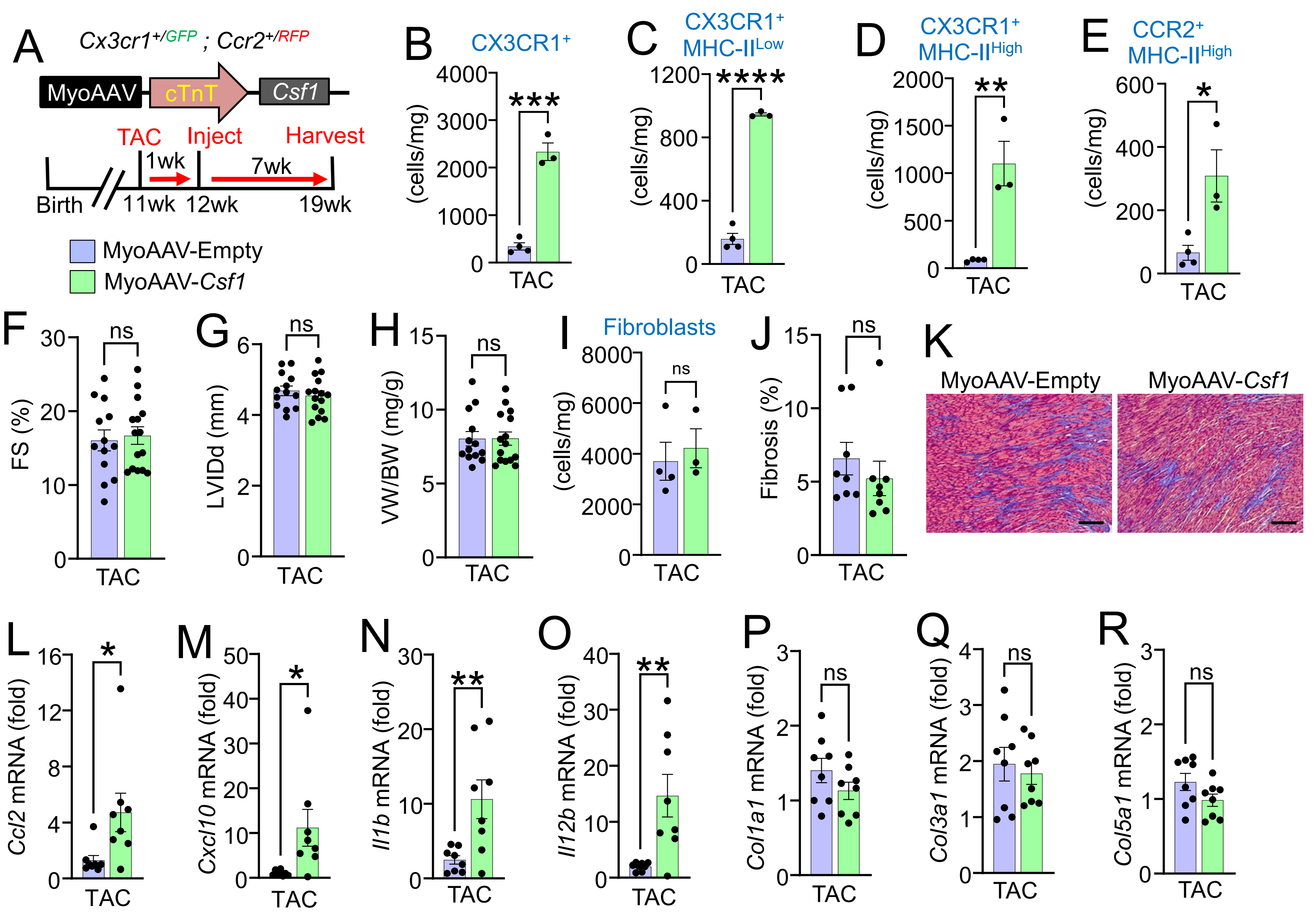

A
Cx3cr1+/GFP ; Ccr2+/RFP
MyoAAV
cTnT
Csf1
TAC
Inject
Harvest
1wk
7wk
Birth
11wk
12wk
19wk
MyoAAV-Empty
MyoAAV-Csf1
B
CX3CR1+
C
CX3CR1+ MHC-IILow
D
CX3CR1+ MHC-IIHigh
E
CCR2+ MHC-IIHigh
(cells/mg)
TAC
***
****
**
*
F
FS (%)
G
LVIDd (mm)
H
VW/BW (mg/g)
I
Fibroblasts
J
Fibrosis (%)
ns
K
MyoAAV-Empty
MyoAAV-Csf1
L
Ccl2 mRNA (fold)
M
Cxcl10 mRNA (fold)
N
Il1b mRNA (fold)
O
Il12b mRNA (fold)
P
Col1a1 mRNA (fold)
Q
Col3a1 mRNA (fold)
R
Col5a1 mRNA (fold)

Supplementary Figure 10: (**A**) Experimental scheme showing adult reporter mice subjected to TAC at 11 weeks of age that were injected with either MyoAAV-Empty or MyoAAV-*Csf1* vector 1 week after TAC and harvested 8 weeks after TAC (7 weeks after MyoAAV delivery). (**B-E**) Flow cytometry quantitation of macrophage subtypes in hearts of these two groups of mice after 8 weeks of TAC stimulation as (**B**) total $CX3CR1^{+}$, (**C**) $CX3CR1^{+}$ $MHC\text{-}II^{Low}$, (**D**) $CX3CR1^{+}$ $MHC\text{-}II^{High}$, and (**E**) $CCR2^{+}$ $MHC\text{-}II^{High}$ macrophages. n=3-4 mice per group, and error bars denote ± SEM, *P<0.05, **P<0.01, ***P<0.001, ****P <0.0001, by two-tailed unpaired Student's t test. (**F-G**) Echocardiography in indicated groups of mice 8 weeks after TAC to assess (**F**) fractional shortening percentage (FS%) and (**G**) left ventricular dimension in diastole. n=13-15 mice per group, and error bars denote ± SEM, by two-tailed unpaired Student's t test. ns, non-significant. (**H**) VW/BW ratio in the indicated two groups of mice 8 weeks after TAC stimulation. n=13-15 mice per group, and error bars denote ± SEM, by two-tailed unpaired Student's t test. ns, non-significant. (**I**) Fibroblast quantification by flow cytometry from hearts of the two indicated groups of mice 8 weeks after TAC. n=3-4 mice per group and, by two-tailed unpaired Student's t test. ns, non-significant. (**J**) Fibrosis quantitation from Masson's trichrome stained cardiac histological sections in the two groups of mice 8 weeks after TAC stimulation. n=8 mice per group, and error bars denote ± SEM, by two-tailed unpaired Student's t test. ns, non-significant. (**K**) Representative cardiac histology images with Masson's trichrome staining for fibrosis (blue) in the two indicated groups 8 weeks after TAC. Scale bar = 100 μm (**L-R**) qRT-PCR analysis of the indicated genes from the hearts of MyoAAV-Empty and MyoAAV-*Csf1* injected mice 8 weeks after TAC. n=8 mice per group, and error bars denote ± SEM, *P<0.05, **P<0.01, by two-tailed unpaired Student's t test.

Supplementary Table 1: Flow cytometry antibody details

Antibody	Clone #	Vendor/Source	Catalogue #
Anti-CD45- BV510	30-F11	BioLegend	103138
Anti-CD11b-Alexa Fluor 700	M1/70	Biolegend	101222
Anti-Ly6G-BV421	1A8	BD Biosciences	562737
Anti-Ly6C-BV605	HK1.4	BioLegend	128036
Anti-CD11c-BV711	N418	BioLegend	117349
Anti-CD64-BV711	X54-5/7.1	BioLegend	139311
Anti-CD64-BV605	X54-5/7.1	BioLegend	139323
Anti-CD64-PE-Cy7	X54-5/7.1	BioLegend	139314
Anti-CD64-APC	X54-5/7.1	BioLegend	139306
Anti-CCR2-APC	475301	R&D Systems	FAB5538A
Anti-MHC-II-PerCP Cy5.5	AF6-120.1	BioLegend	116416
Anti-Timd4- PE-Cy7	RMT4-54	BioLegend	130010
Anti-CD3-APC	17A2	BioLegend	100236
Anti-CD4-PerCP Cy5.5	RM4-4	BioLegend	116011
Anti-CD8a-BV421	53-6.7	BioLegend	100737
Anti-CD19-BV605	6D5	BioLegend	115520
Anti-NK1.1-PE Cy7	PK136	BioLegend	108714
Anti-CD31-BV421	390	BioLegend	102424
Anti-CD31-BV605	390	BioLegend	102427
Anti-Feeder Cells-APC	mEF-SK4	Miltenyi Biotec	130-120-802

Supplementary Table 2: Immunofluorescence antibody details

Antibody	Vendor/Source	Catalogue #
Anti-GFP	Abcam	ab13970
Anti-RFP	OriGene Technologies	AB1140-100
Anti-CD68	Abcam	ab53444
Anti-PDGFRα	R&D Systems	AF1062
Anti-Vimentin	Abcam	Ab45939
Anti-Periostin	Novus Biologicals	NBP1-30042
Donkey Anti-Chicken-Alexa Fluor 488	Jackson ImmunoResearch	703-545-155
Donkey Anti-Goat-Alexa Fluor 594	Jackson ImmunoResearch	705-585-147
Donkey Anti-Rat- Alexa Fluor 647	Jackson ImmunoResearch	712-605-153
Donkey Anti-Goat- Alexa Fluor 647	Jackson ImmunoResearch	705-605-147
Donkey Anti-Rabbit Alexa Fluor 647	Jackson ImmunoResearch	711-605-152

Supplementary Table 3: List of qRT-PCR primer sequences used in the study

Gene	Forward	Reverse
Il6	5’ TCCAGTTGCCTTCTTGGGAC 3’	5’ GTGTAATTAAGCCTCCGACTTG 3’
Il1b	5’ AGTTGACGGACCCCAAAAG 3’	5’ AGCTGGATGCTCTCATCAGG 3’
Tnf	5’ TCTTCTCATTCCTGCTTGTGG 3’	5’ GGTCTGGGCCATAGAACTGA 3’
Ccl2	5’ CATCCACGTGTTGGCTCA 3’	5’ GATCATCTTGCTGGTGAATGAGT 3’
Cxcl10	5’ GCTGCCGTCATTTTCTGC 3’	5’ TCTCACTGGCCCGTCATC 3’
Il12b	5’ AAGGAACAGTGGGTGTCCAG 3’	5’ GTTAGCTTCTGAGGACACATCTTG 3’
Tgfb1	5’ TGGAGCAACATGTGGAACTC 3’	5’ GTCAGCAGCCGGTTACCA 3’
Col1a1	5’ CATGTTCAGCTTTGTGGACCT 3’	5’ GCAGCTGACTTCAGGGATGT 3’
Col3a1	5’ TCCCCTGGAATCTGTGAATC 3’	5’ TGAGTCGAATTGGGGAGAAT 3’
Col5a1	5’ CTACATCCGTGCCCTGGT 3’	5’ CCAGCACCGTCTTCTGGTAG 3’
Fn1	5’ CGGAGAGAGTGCCCCTACTA 3’	5’ CGATATTGGTGAATCGCAGA 3’
Hprt	5’GCCCTTGACTATAATGAGTACTT CAGG 3’	5’ TTCAACTTGCGCTCATCTTAGG 3’
18S rRNA	5’ TTTCTCGATTCCGTGGGTGG 3’	5’ TCAATCTCGGGTGGCTGAAC 3’
